# Targeted multimodal theranostics *via* biorecognition controlled aggregation of metallic nanoparticle composites†

**DOI:** 10.1039/c6sc01463a

**Published:** 2016-05-04

**Authors:** Xi-Le Hu, Yi Zang, Jia Li, Guo-Rong Chen, Tony D. James, Xiao-Peng He, He Tian

**Affiliations:** a Key Laboratory for Advanced Materials & Institute of Fine Chemicals, East China University of Science and Technology 130 Meilong Rd. Shanghai 200237 PR China xphe@ecust.edu.cn; b National Center for Drug Screening, State Key Laboratory of Drug Research, Shanghai Institute of Materia Medica, Chinese Academy of Sciences 189 Guo Shoujing Rd. Shanghai 201203 PR China jli@simm.ac.cn; c Department of Chemistry, University of Bath Bath BA2 7AY UK

## Abstract

We have developed a theranostic nanocomposite of metallic nanoparticles that uses two distinct fluorescence mechanisms: Förster Resonance Energy Transfer (FRET) and Metal-Enhanced Fluorescence (MEF) controlled by ligand–receptor interaction. Supramolecular assembly of the fluorophore-labeled glycoligands to cyclodextrin-capped gold nanoparticles produces a nanocomposite with a quenched fluorescence due to FRET from the fluorophore to the proximal particle. Subsequently, interaction with a selective protein receptor leads to an aggregation of the composite, reactivating the fluorescence by MEF from the distal metallic particles to fluorophores encapsulated in the aggregates. The aggregation also causes a red-shift in absorbance of the composite, thereby enhancing the production of reactive oxygen species (ROS) on red-light irradiation. Our nanocomposite has proven suitable for targeted cancer cell imaging as well as multimode therapy using both the photodynamic and drug delivery properties of the composite.

## Abstract 2

We show that two distinct mechanisms, FRET and MEF, of metallic nanoparticles can be finely tuned by a ligand–receptor interaction, producing a nanocomposite with an enhanced ROS production for targeted imaging and multimodal therapy.

## Graphical abstract

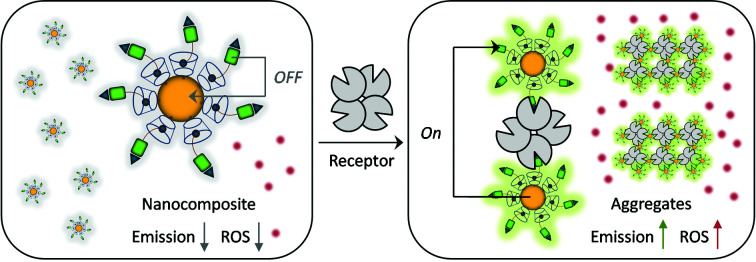

## Introduction

Metallic nanoparticles are attractive nanomaterials that have found applications in a diverse range of research fields. In particular, because of their unique optical properties, these materials have been employed for the diagnosis and photodynamic therapy of human diseases. For example, gold nanoparticles (AuNPs) are used for the naked-eye detection of biomolecules and pathogens due to the sensitive colorimetric change observed upon aggregation.^1–9^ They have also been used as photoluminescent agents (gold nanoclusters)^10^ for cellular and *in vivo* imaging. More recently, using the localized surface plasmon resonance (LSPR) of AuNPs, novel sensors have been developed using Raman spectroscopy.^11–14^ It is also well known that long-wavelength irradiation of AuNPs can promote the production of reactive oxygen species (ROS) to kill cancer cells and pathogens in a photodynamic manner.^15,16^

AuNPs can tune the emission of fluorophores when the distance between the two species is adjusted. For example, the Förster Resonance Energy Transfer (FRET) mechanism, where the AuNP serves as an energy acceptor and a proximal fluorophore as the energy donor, may result in a quenched fluorescence of the latter.^17^ However, a longer distance between the AuNP and fluorophore can cause an enhancement of fluorescence of the latter *via* the metal-enhanced fluorescence (MEF) mechanism.^18^ With this research we demonstrate that these two distinct mechanisms can be finely tuned by ligand–receptor recognition, in order to develop a nanocomposite for targeted theranostics.

**Fig. 1:**
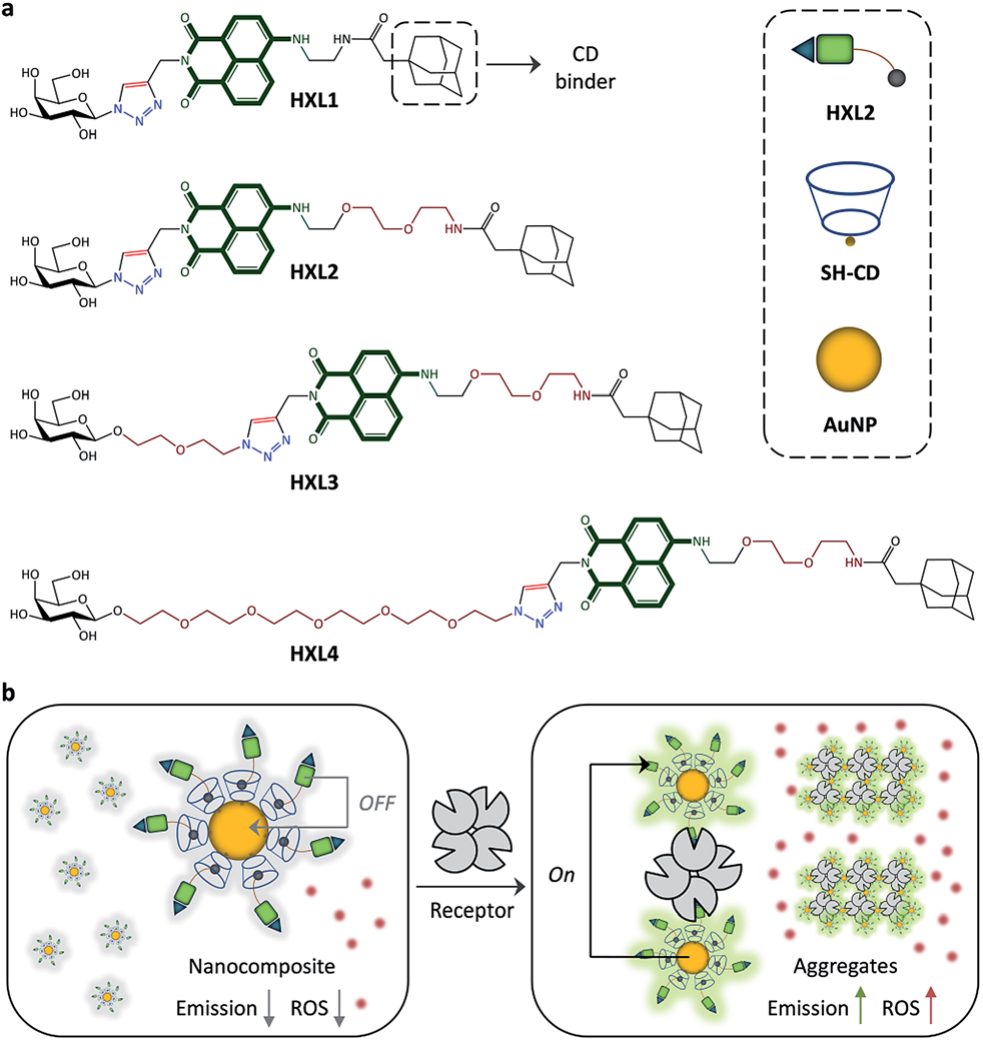
(a) Structure of the fluorophore-labeled glycoligands (HXLs) with adamantane as a cyclodextrin binder and cartoon of HXL2, SH-cyclodextrin (SH-CD) and gold nanoparticle (AuNP). (b) Schematic illustration of the nanocomposite based on the host–guest interaction between CD-AuNP and HXL2 with quenched fluorescence, and enhancement of both fluorescence and reactive oxygen (ROS) production by ligand–receptor recognition.

Shown in Fig. 1a are the structures of the fluorophore-labeled ligands. A glycoligand (galactose) was coupled to naphthalimide using a click reaction,^19,20^ followed by introduction of adamantane to the dye moiety for the coating of a cyclodextrin-attached AuNP (CD-AuNP).^21^ Four analogues with different alkyl chain lengths between dye and adamantane (HXL1*vs.*HXL2) or between dye and glycoligand (HXL2*vs.*HXL3*vs.*HXL4) were synthesized for evaluation of the structure–activity relationship. These compounds were then coated onto the CD-AuNP using the adamantane-CD host–guest interaction, in order to produce nanocomposites (Fig. 1b). While quenched fluorescence is observed because of FRET from the naphthalimide to the proximal AuNP, subsequent aggregation of the composites by selective receptor protein interactions then enhances the fluorescence by MEF from distal AuNPs to the fluorophores encapsulated in the protein aggregate (Fig. 1b). Meanwhile, the aggregation also causes a red-shift in absorbance of the nanocomposite, thereby enhancing the ability to produce ROS upon red-light irradiation.

**Fig. 2:**
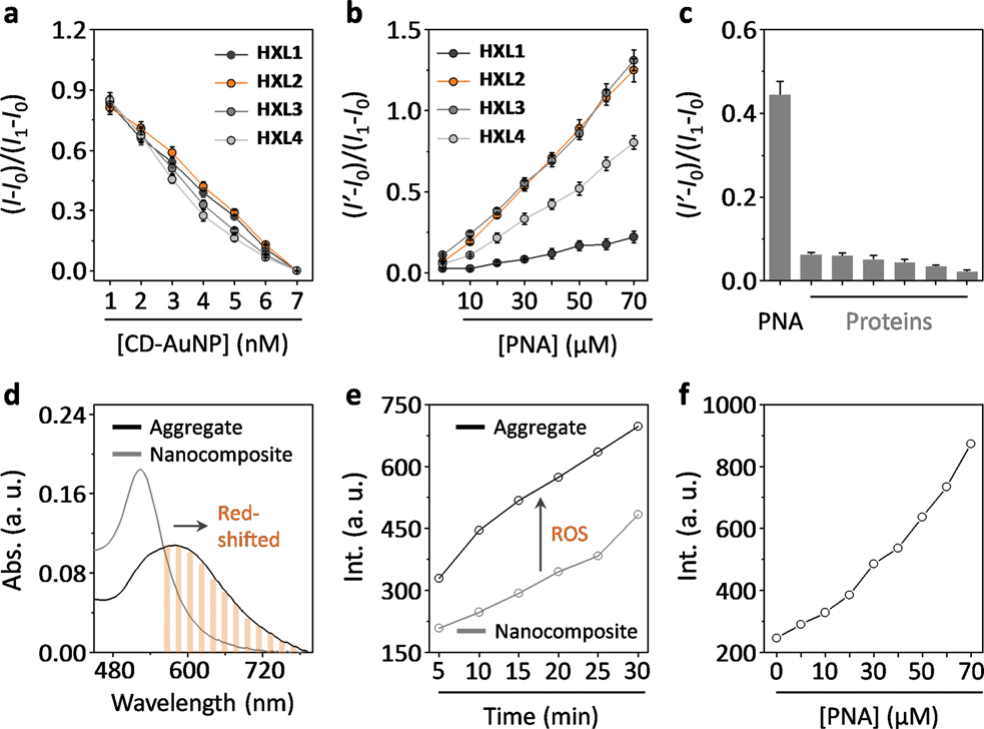
(a) Concentration-dependent fluorescence quenching of HXLs (0.56 μM) in the presence of increasing CD-AuNP. (b) Concentration-dependent fluorescence recovery of HXLs@CD-AuNP (0.56 μM/7 nM) in the presence of increasing peanut agglutinin (PNA). (c) Fluorescence enhancement of HXL2@CD-AuNP (0.56 μM/7 nM) in the presence of different proteins (30 μM, from left to right: pepsin, wheat germ agglutinin, ribonuclease, bovine serum albumin, concanavalin A and *Lens culinaris* lectin) (*I*_1_, *I*_0_, *I* and *I*′ are the fluorescence intensity of HXL, HXL@CD-AuNP, HXL with CD-AuNP of a certain concentration and HXL@CD-AuNP with a protein of a certain concentration, respectively). (d) UV-vis absorbance of HXL2@CD-AuNP (0.56 μM/7 nM) in the absence and presence of PNA (70 μM). (e) Reactive oxygen species (ROS) production after irradiation (600 nm) of HXL2@CD-AuNP (0.56 μM/7 nM, composite) in the absence and presence of PNA (30 μM, aggregate) with time. (f) Reactive oxygen species (ROS) production of HXL2@CD-AuNP (0.56 μM/7 nM) with increasing PNA after irradiation (600 nm) for 10 min.

Firstly, we tested the fluorescence quenching of HXLs after supramolecular assembly with CD-AuNPs in a Tris–HCl buffer solution (0.01 M, pH 7.4). The results indicated that the fluorescence of all of the naphthalimides quenched in a concentration-dependent manner with increasing CD-AuNP (Fig. 2a and S1a†), suggesting that the compounds are immobilized in proximity to the particle surface. The overlapping emission band of the naphthalimide and absorbance band of the AuNP suggests that FRET is the cause of the fluorescence quenching (Fig. S2†). Subsequently, we observed that the addition of a selective galactose receptor, peanut agglutinin (PNA), gradually enhances the fluorescence of the nanocomposites with different recovery rates (Fig. 2b and S1b†). While only a minimal difference in quenching was observed for HXL1 and HXL2 (Fig. 2a), the fluorescence recovery of the latter was much stronger than the former (Fig. 2b). However, slight chain elongation displayed little effect on the fluorescence (HXL3*vs.*HXL2), whereas a more distant coupling between ligand and fluorophore decreased the rate of fluorescence enhancement (HXL4*vs.*HXL2). We also determined that the enhancement was specific for PNA over a range of unselective proteins (Fig. 2c and S3†). The observed enhancement of fluorescence is probably the result of MEF, which could occur in the protein–nanocomposite aggregate matrix, from the distal AuNPs to the fluorophores. Our observations are in agreement with the previously reported MEF-based biosensing systems.^22–24^ Our results also suggest that optimization of the molecular length of the compounds can enhance the MEF efficiency within the aggregates.

To substantiate the FRET and MEF mechanisms, we have carried out additional fluorescence life-time measurements. On the one hand, with increasing CD-AuNP a sequential decrease of lifetime of HXL2 is observed, indicating energy transfer from the compound to nanoparticles (Fig. S4a†).^25^ While on the other hand, according to a previous report^24^ we determined the radiative (*k*_r_) and nonradiative kinetic (*k*_nr_) parameters for HXL2, HXL2@CD-AuNP and HXL2@CD-AuNP in the presence of PNA. While, a slight change in *k*_nr_ of HXL2 upon association with CD-AuNP and interaction with PNA is probably the result of small disruptions of the nanoparticles, the increased *k*_r_ of the HXL2@CD-AuNP nanocomposite in the presence of PNA (6.1 × 10^−7^ S^−1^) with respect to the nanocomposite itself (1.1 × 10^−7^ S^−1^) suggests that the lectin-induced aggregation of nanoparticles increases the electromagnetic resonance coupling. Therefore, corroborating a MEF fluorescence enhancement mechanism.^24^

Next, we determined that the absorbance band of the composite was red-shifted after aggregation with PNA (Fig. 2d). This leads to a drastic increase in the production of ROS upon red-light irradiation (600 nm) with time (Fig. 2e), as measured by a ROS trapper.^26^ The ROS signal was also observed to intensify gradually with increasing PNA (Fig. 2f). These results indicate that, in addition to the enhanced emission, the aggregation also facilitates the ability of the nanocomposite to produce ROS upon red-light irradiation.

**Fig. 3:**
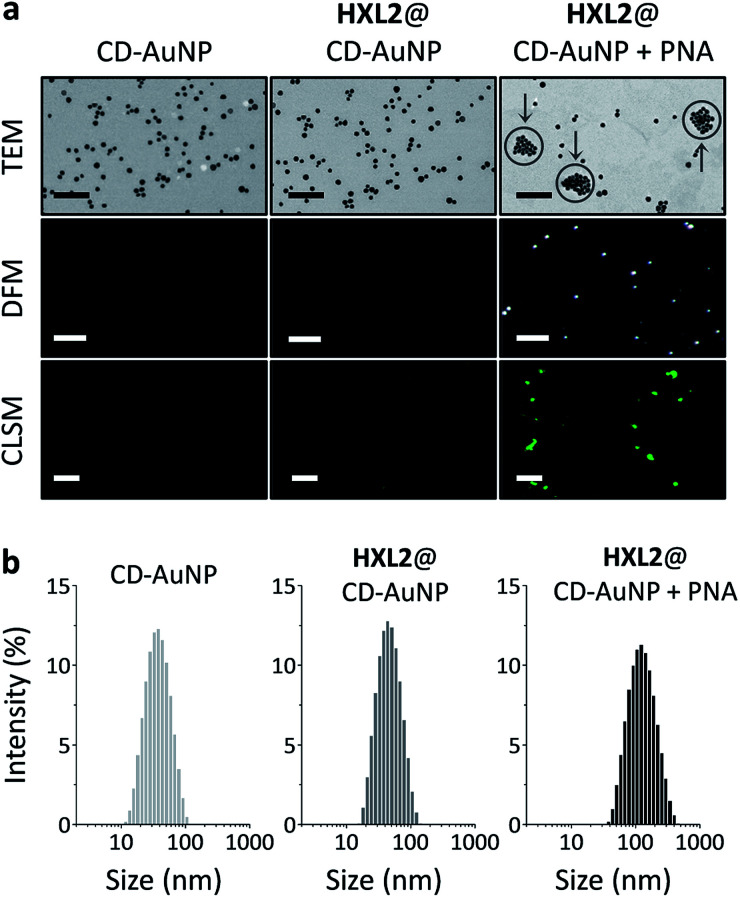
(a) Transmission electron microscopy (TEM, scale bar = 200 nm, arrows indicate aggregated particles), dark-field microscopy (DFM, scale bar = 20 μm) and confocal laser scanning microscopy (CLSM, scale bar = 10 μm) of CD-AuNP (7 nM) and HXL2@CD-AuNP (0.56 μM/7 nM) without or with PNA (70 μM). (b) Dynamic light scattering of CD-AuNP (7 nM) and HXL2@CD-AuNP (0.56 μM/7 nM) without or with PNA (30 μM).

To further characterize the aggregation of the nanocomposite, a series of microscopic techniques were employed (Fig. 3). With transmission electron microscopy (TEM) we observed that bare CD-AuNP and the HXL2@CD-AuNP composite were monodispersed particles, whereas addition of PNA caused particle aggregation (Fig. 3a). The morphological change is in agreement with data obtained from dynamic light scattering (the particle size of the composite increased sharply with added PNA) (Fig. 3b). Dark-field microscopy (DFM) was also used to characterize the aggregation, considering that an intensified scattering can be recorded with large AuNP aggregates due to coupled plasmonic oscillations of the aggregated AuNPs.^11–14^ With minimal signals for CD-AuNP and the HXL2@CD-AuNP composite, we observed strong scattering spots for the PNA-aggregated particles. Similarly, confocal laser scanning microscopy (CLSM) corroborated that the quenched fluorescence emission of HXL2@CD-AuNP composite could be recovered in the presence of PNA (Fig. 3a). These data support the aggregation as well as the resulting fluorescence enhancement of the nanocomposite.

**Fig. 4:**
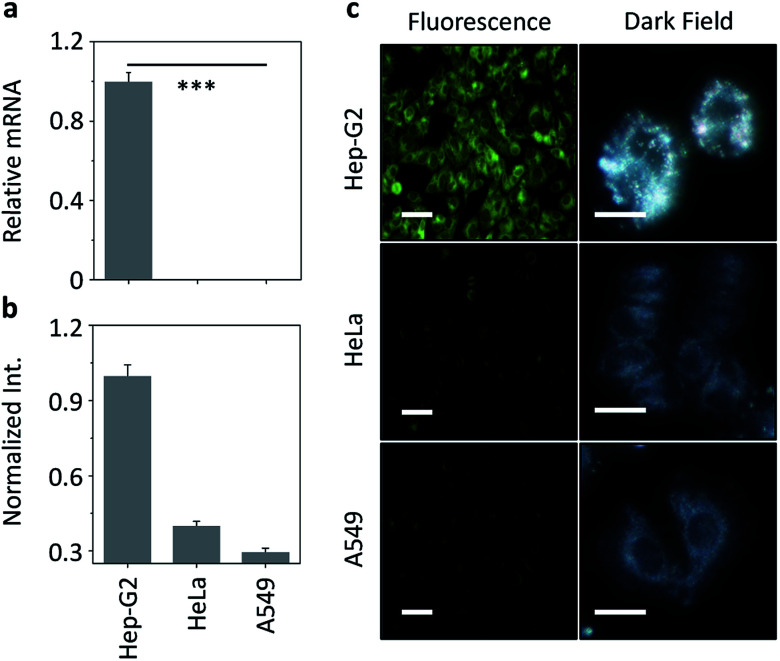
(a) Normalized mRNA level of asialoglyprotein receptor determined by real-time quantitative polymerase chain reaction for Hep-G2 (human hepatoma), HeLa (human cervical cancer) and A549 (human lung cancer) cells (***P < 0.001). (b) Fluorescence quantification of different cells after treatment with HXL2@CD-AuNP (10 μM/100 nM). (c) Fluorescence and dark field imaging (scale bars: 20 μm; excitation channel: 410–430 nm; emission channel: 460–540 nm) of different cells after treatment with HXL2@CD-AuNP (10 μM/100 nM).

We then set out to evaluate our nanocomposite for receptor-targeted cell imaging. A hepatoma cell line (Hep-G2) that expresses a transmembrane galactose receptor (the asialoglycoprotein receptor – ASGPr) and control cell lines (human cervical cancer – HeLa and human lung cancer – A549) with minimal ASGPr expression were used.^27^ The ASGPr expression level was examined by real-time quantitative polymerase chain reaction (RT-qPCR) (Fig. 4a). Treatment of HXL2@CD-AuNP with the cells only led to a fluorescence production in Hep-G2 cells, but not in the control cells, as determined by both fluorescence quantification (Fig. 4b) and imaging (Fig. 4c). To evaluate whether the fluorescence was dependent on ASGPr–HXL2 recognition, the following assays were also carried out: (1) knockdown of ASGPr largely decreased the nanocomposite fluorescence produced in Hep-G2 (Fig. S5†), and (2) preincubation with increasing amounts of free d-galactose and Hep-G2 also gradually suppressed the fluorescence (Fig. S6†). Meanwhile, the nanocomposite with an increasing HXL2 concentration did not show toxicity to Hep-G2 and mouse embryonic fibroblast cell lines (Fig. S7†). The fact that both knockdown of ASGPr and competition by free galactose suppressed the fluorescence suggests a receptor-dependent interaction of the nanocomposite with Hep-G2.

We also used DFM to analyze the interaction of HXL2@CD-AuNP nanoemsemble with the cells (Fig. 4c). We observed strong AuNP scattering in Hep-G2 cells, where the majority of the particles were aggregated (Fig. 4c). This is in accordance with the fluorescence detected in Hep-G2 cells, suggesting that the intracellular aggregation of the nanocomposites was mediated by ASGPr. However, minimal scattering signals were recorded for the control cells (HeLa and A549) without ASGPr expression. These cellular assays suggest the ability of the nanocomposite developed for targeted cell imaging by receptor-mediated intracellular aggregation.

**Fig. 5:**
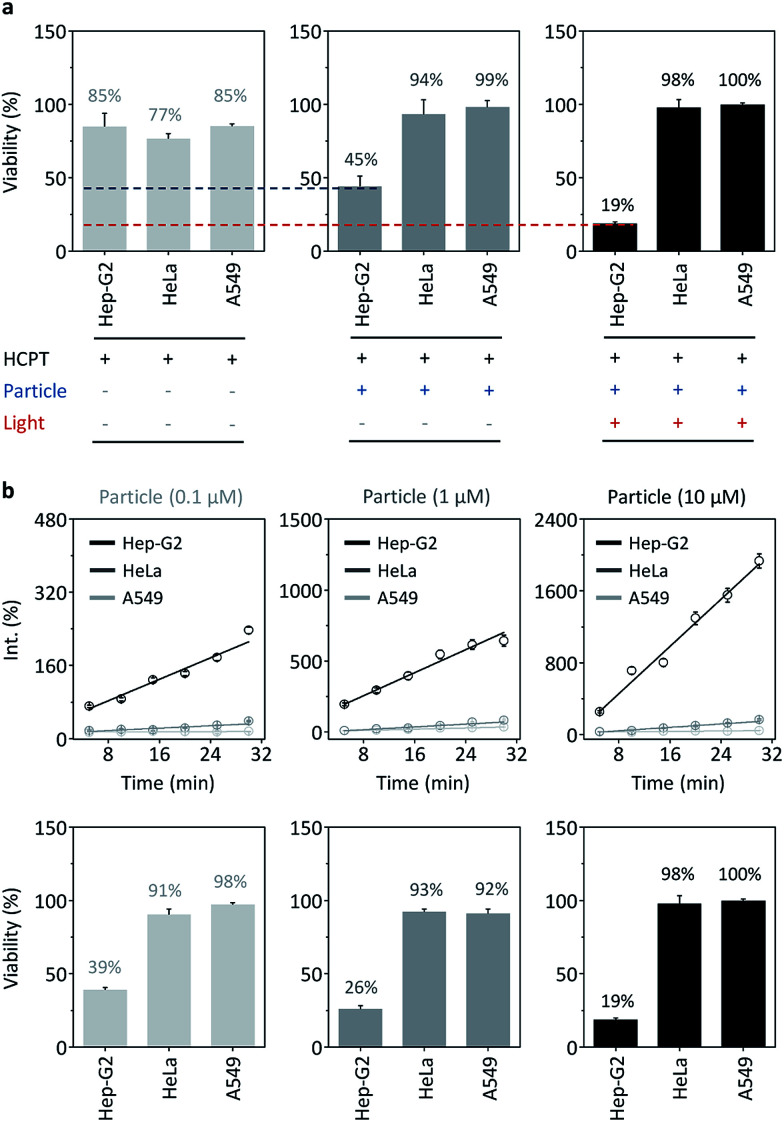
(a) Cell viability of Hep-G2, HeLa and A549 after treatment with hydroxycamptothecin (HCPT, 1 μM), HCPT@HXL2@CD-AuNP (1 μM/10 μM/100 nM, particle) and HCPT@HXL2@CD-AuNP with red-light irradiation (600 nm, 30 min). (b) Concentration/time-dependent reactive oxygen species (ROS) production of the particle with red-light irradiation after incubation with different cells and a resulting cell viability after 30 min irradiation.

Subsequently, the therapeutic potential of the nanocomposite was evaluated using both the photodynamic^15,16^ as well as drug delivery properties of the AuNPs.^28–30^ We first mixed an anticancer drug, hydroxycamptothecin (HCPT), with the nanocomposite. The cell viability assay showed that while a short-term (15 min) incubation of HCPT alone with different cancer cells (Hep-G2, HeLa and A549) resulted in a slight cytotoxic effect probably because of insufficient internalization of the drug by the cells (Fig. 5a), loading of the drug with the nanocomposite significantly enhanced the toxicity for Hep-G2, but not for the control cells (Fig. 5a). This suggests that the nanocomposite is able to quickly deliver the drug to Hep-G2 probably by receptor-mediated endocytosis, while also preventing the unselective uptake of the drug by other cells (since the cell viability of HeLa and A549 treated with the nanocomposite is higher than those treated with drug alone).

Given that the aggregation of the nanocomposite in cells enhances the production of ROS, we also irradiated the cells pretreated with the nanocomposite–drug hybrid with red-light (600 nm). In addition to the cytotoxicity of the drug, we observed that the irradiation further suppressed the cell viability of Hep-G2, but not that of the control cells (Fig. 5a). This suggests that the photodynamic therapy is similarly target-specific. To evaluate the photodynamic therapy, we detected the ROS production of nanocomposite for different cells. We observed that ROS was produced selectively with Hep-G2 cells in a concentration dependent manner over the control cells (Fig. 5b). The ROS production resulted in a concentration-dependent cell death for Hep-G2, but not for HeLa and A549 (Fig. 5b). These data suggest a multimode therapeutic potential (drug loading and photodynamic ROS production, both of which function predominantly for receptor-rich cells) of the nanocomposite for targeted disease theranostics.

## Conclusions

To summarize, we developed a unique nanocomposite based on the switch of two distinct mechanisms of AuNPs. The composite in its fluorescence-quenched form displays a much more enhanced emission upon aggregation *via* a selective receptor protein interaction. This aggregation also facilitates ROS production upon red-light irradiation because of a red shift of the absorbance band. As a proof-of-concept, the nanocomposite has been demonstrated to work as a receptor-targeting cell imaging and multimode theranostic system, using both the drug carrying and photodynamic properties of the nanocomposite. We believe that our research paves the way for the development of a diverse array of fluorogenic, therapeutic nanomaterials based on the diversity of available metallic nanoparticles.

## Supplementary Material

SC-007-C6SC01463A-s001
