# Supplementary material for: Targeted multimodal theranostics via biorecognition controlled aggregation of metallic nanoparticle composites
Source: Chem Sci. 2016 May 4;7(7):4004–8. doi: 10.1039/c6sc01463a (PMC6013785; doi:10.1039/c6sc01463a)
Supplement: SC-007-C6SC01463A-s001 [file SC-007-C6SC01463A-s001.pdf]

Electronic Supplementary Material (ESI) for Chemical Science.
This journal is © The Royal Society of Chemistry 2016

Supporting Information

Targeted Multimodal Theranostics via Biorecognition Controlled Aggregation of Metallic Nanoparticle Composites

Xi-Le Hu,[1] Yi Zang,[2] Jia Li,[2]* Guo-Rong Chen,[1] Tony D James,[3] Xiao-Peng He,[1]* and He Tian

[1]*Key Laboratory for Advanced Materials & Institute of Fine Chemicals, East China University of Science and Technology, 130 Meilong Rd., Shanghai 200237, PR China*
[2]*National Center for Drug Screening, State Key Laboratory of Drug Research, Shanghai Institute of Materia Medica, Chinese Academy of Sciences, 189 Guo Shoujing Rd., Shanghai 201203, PR China*
[3]*Department of Chemistry, University of Bath, Bath, BA2 7AY, UK*

Contents list

S1. Additional figures
S2. Experimental section
S3. Spectral copies of new compounds
S4. Additional references

S1. Additional figures

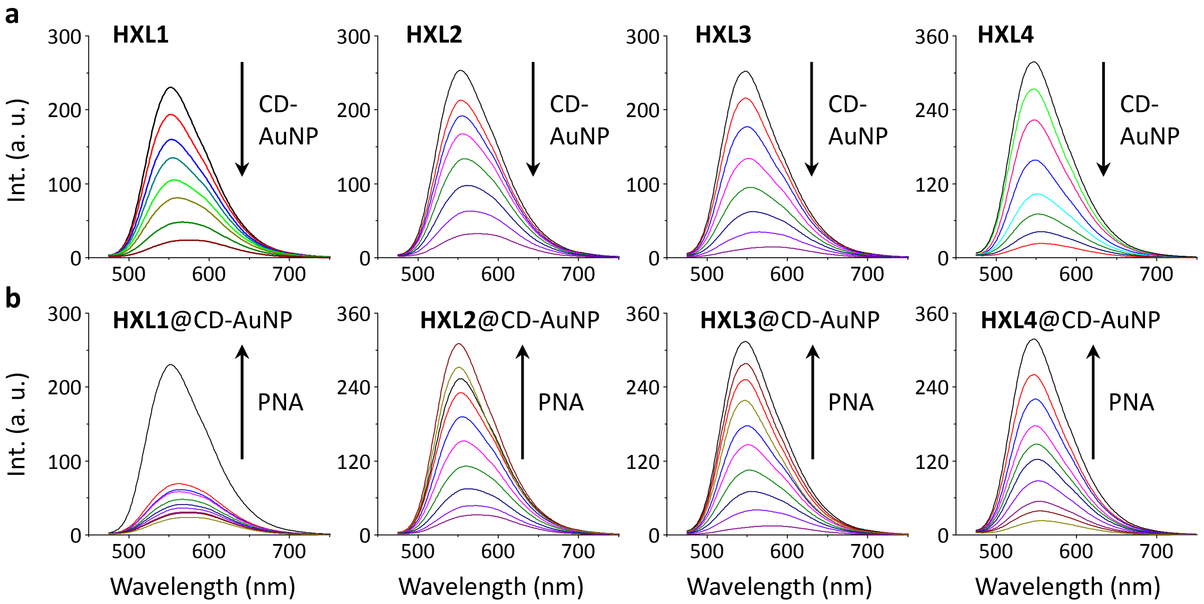

Figure S1. Fluorescence (Int. = intensity) titration of (a) **HXL**s (0.56 μM) in the presence of increasing CD-AuNP (0-7 nM) and (b) **HXL**s@CD-AuNP (0.56 μM/7 nM) in the presence of increasing peanut agglutinin (PNA, 0-70 μM) in Tris-HCl buffer (0.01 M, pH 7.4). Excitation wavelength = 447 nm.

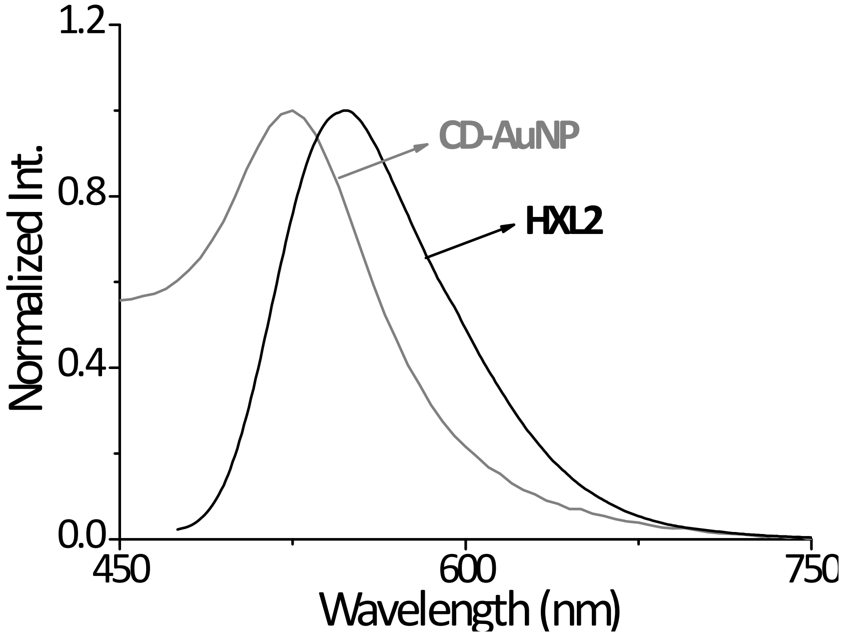

Figure S2. Stacked absorbance spectrum of CD-AuNP and emission spectrum of **HXL2** in Tris-HCl buffer (0.01 M, pH 7.4). Excitation wavelength = 447 nm.

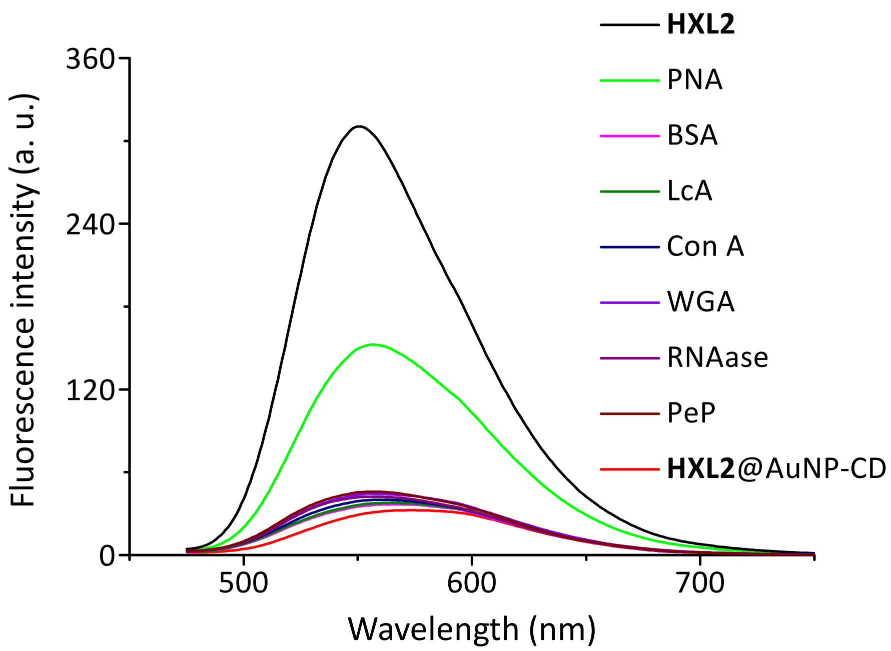

Figure S3. Stacked fluorescence spectra of **HXL**s@CD-AuNP (0.56 μM/7 nM) in the absence and presence of different proteins (30 μM) (PNA = Peanut agglutinin; BSA = Bovine serum albumin; LcA = *Lens culinaris* lectin; Con A = Concanavalin A; WGA = Wheat germ agglutinin; RNase = Ribonuclease; Pep = Pepsin) in Tris-HCl buffer (0.01 M, pH 7.4). Excitation wavelength = 447 nm.

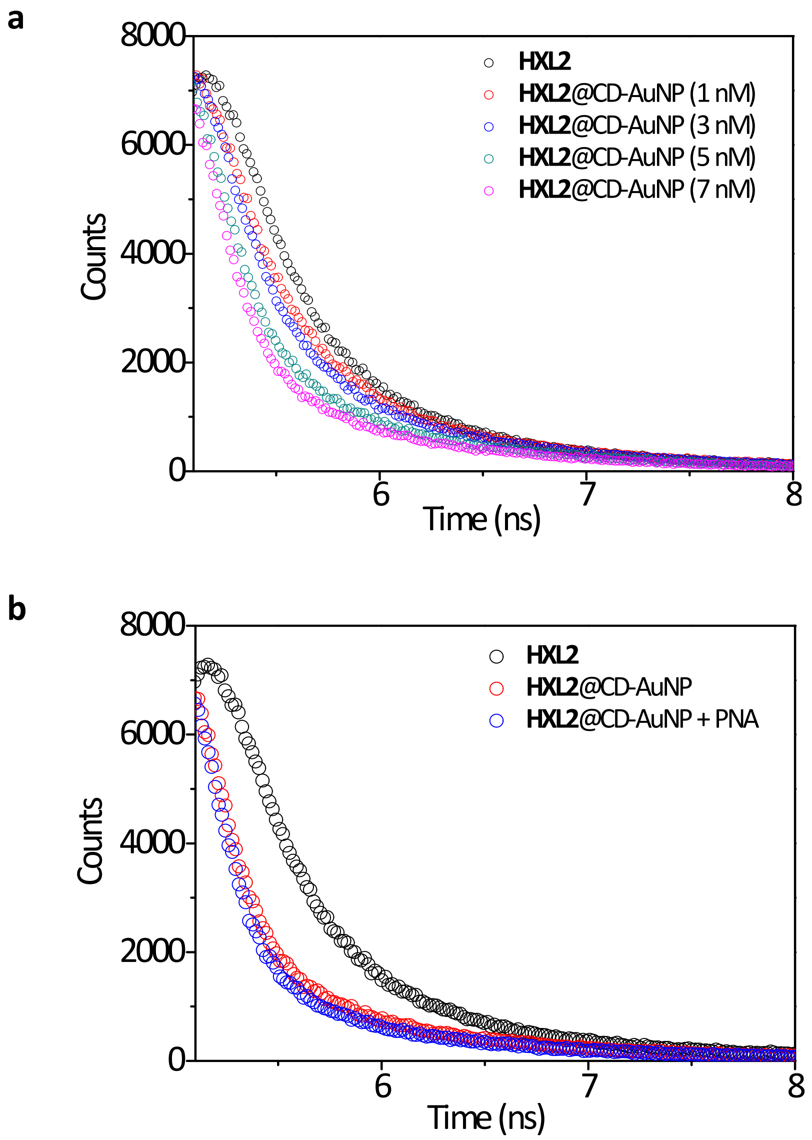

Sample	φ_F	Γ_{ave} (ns)	K_r (S^{-1})	K_{nr} (S^{-1})
**HXL2**	0.115	6.46	1.7×10^7	1.3×10^8
**HXL2**@CD-AuNP	0.043	4.09	1.1×10^7	2.3×10^8
**HXL2**@CD-AuNP + PNA	0.195	3.22	6.1×10^7	2.5×10^8

Figure S4. Excited-state lifetime decay plots of (a) **HXL2** (0.56 μM) in the presence of increasing CD-AuNP and (b) **HXL2** (0.56 μM) and **HXL2**@CD-AuNP (0.56 μM/7 nM) without or with PNA (70 μM) (the table details the quantum yield [φ_F], average lifetime [Γ_{ave}], radiative [k_r] and nonradiative [k_{nr}] parameters).

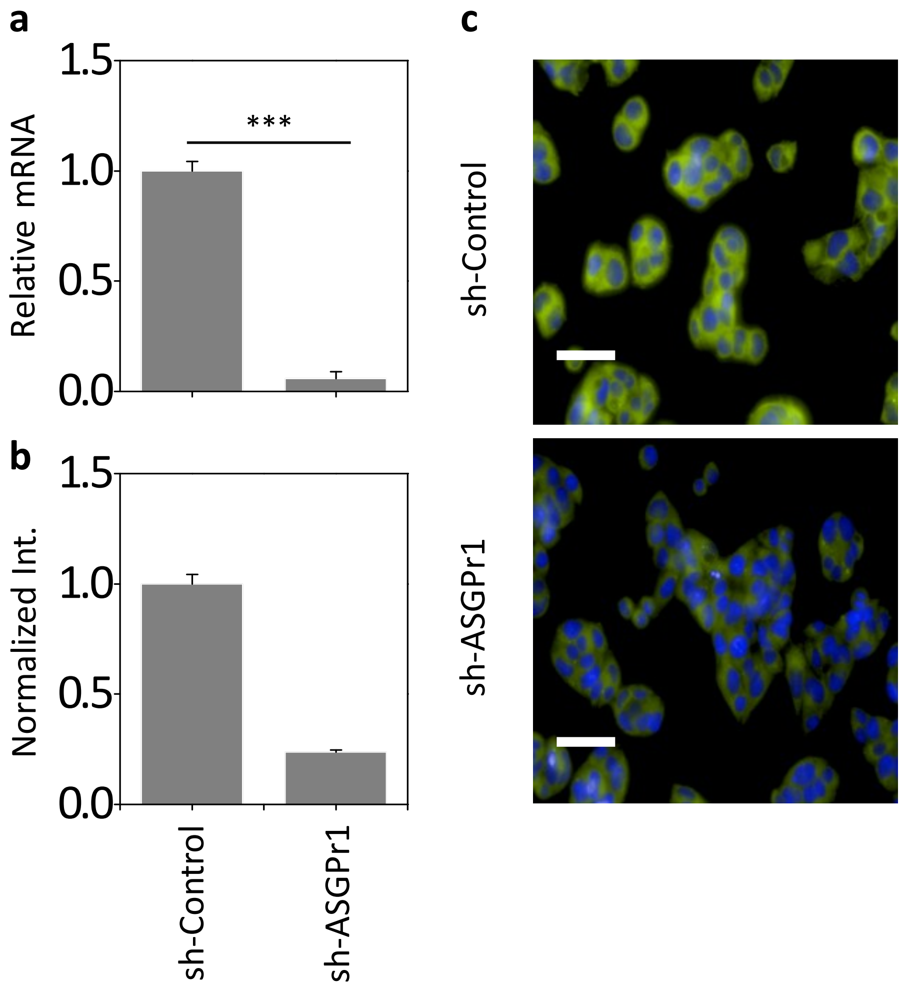

Figure S5. (a) Normalized mRNA level of asialoglyprotein receptor determined by real-time quantitative polymerase chain reaction (RT-q-PCR) for sh-Control (Hep-G2 cells transfected with a scrambled sh-RNA) and sh-ASGPr1 (Hep-G2 cells transfected with sh-ASGPr1) (****P*< 0.001). (b) Fluorescence quantification and (c) imaging of the two cells after treatment with **HXL2**@CD-AuNP (10 μM/100 nM) (scale bar: 20 μm; excitation channel = 410-430 nm; emission channel: 460-540 nm).

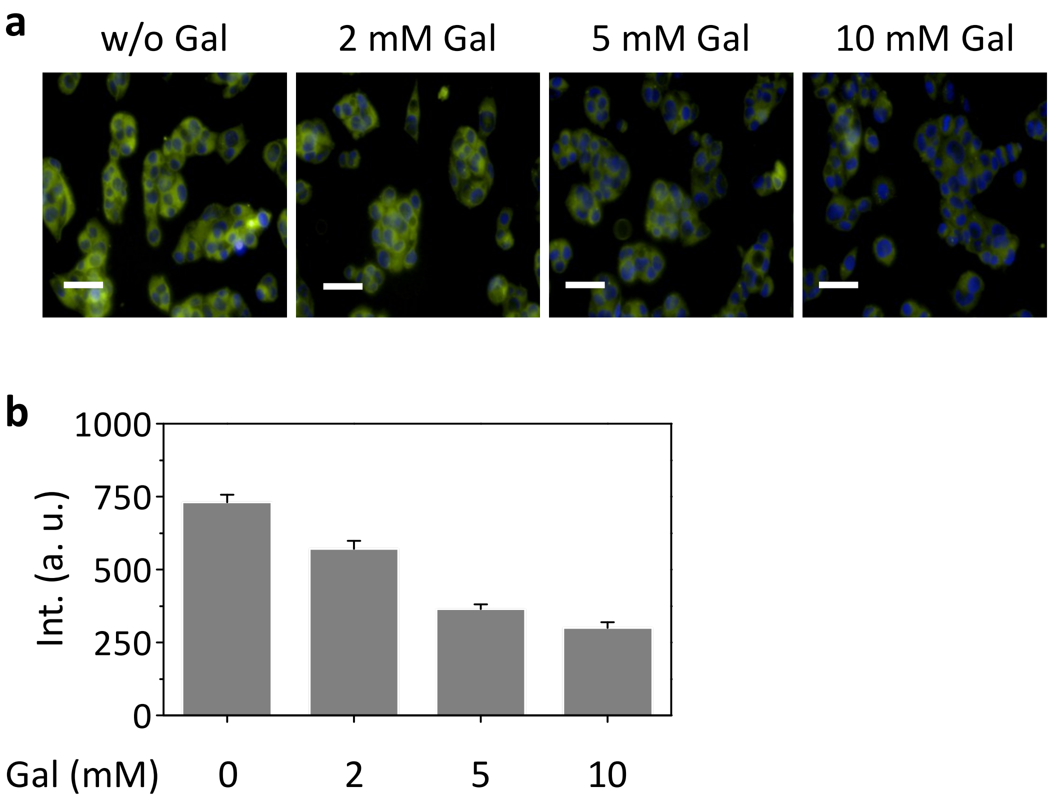

Figure S6. (a) Fluorescence imaging and (b) quantification of Hep-G2 cells pretreated with different concentrations of free D-galactose with **HXL2**@CD-AuNP (10 μM/100 nM) (scale bar: 20 μm; excitation channel = 410-430 nm; emission channel: 460-540 nm).

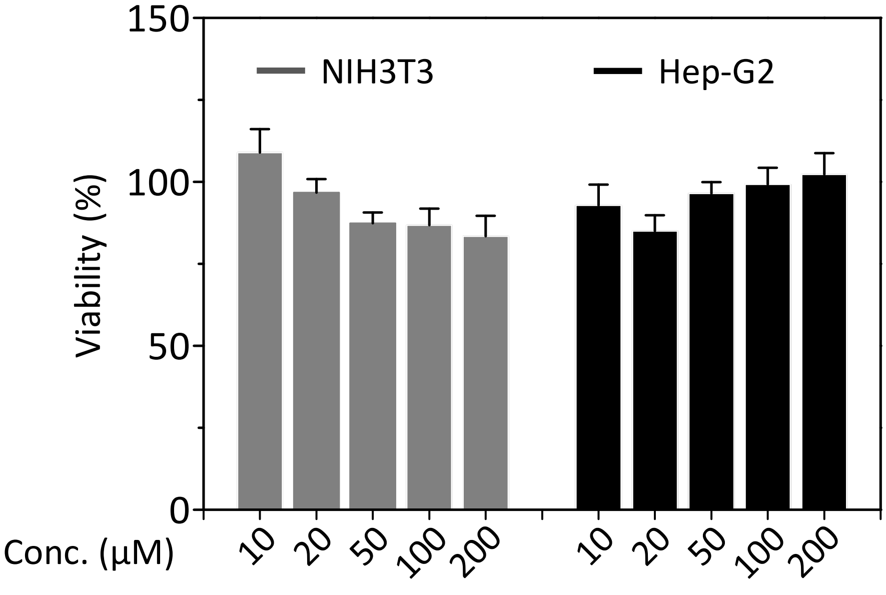

Figure S7. Cell viability of Hep-G2 (human hepatoma) and NIH3T3 (mouse embryonic fibroblast) after treatment with increasing **HXL2** mixed with CD-AuNP (10 nM).

S2. Experimental section

General. All purchased chemicals and reagents are of analytical grade. Hydrogen tetrachloroaurate (III) hydrate ($HAuCl_4 \cdot XH_2O$, 99.9%-Au) was purchased from J&K Chemical. Sodium citrate was obtained from J&K Chemical. Mercapto-β-cyclodextrin (β-CD-SH) was purchased from Shandong Zhiyuan Bio-technology Ltd (China). Proteins were purchased from Sigma-Aldrich. ^{1}H NMR and ^{13}C NMR spectra were recorded on a Bruker AM 400 MHz spectrometer with tetramethylsilane (TMS) as internal reference. Absorption spectra were measured on a Varian Cary 500 UV-Vis spectraphotometer. High resolution mass spectra (HRMS) were recorded with a Waters Micromass LCT mass spectrometer. Transmission electron microscopy (TEM) images were obtained on a JEOL 100CX transmission electron microscope operating at an accelerating bias voltage of 100 kV. Dynamic light scattering (DLS) was carried out on a Horiba LB-550 Dynamic Light Scattering Nano-Analyzer. High Performance Liquid Chromatography (HPLC) was measured on a Shimadzu Prominence Series equipment.

Preparation of nanocomposites. To prepare the nanocomposites, a solvent displacement method was carried out using water and dimethylsulfoxide. A dimethylsulfoxide solution of **HXL**s (1.5 μM) and an aqueous solution of mono-6-thiol-β-CD capped AuNP (7 nM) prepared according to a previous report[1] were mixed and stirred vigorously. **HXL** molecules with an adamantine tail could be entrapped to the hydrophobic cavity of CD due to hydrophobic interactions. The mixture was then centrifuged at 8000 rpm for 20 min to remove residual **HXL** molecules. Then, the **HXL**@CD-AuNP solution was further washed with Tris-HCl buffer (0.01 M, pH 7.4) and centrifuged. This step was repeated three times. The hydrocamptothecin (HCPT)-nanocomposite drug delivering system was prepared by the same protocol.

Cell culture. Hep-G2 and HeLa cells were maintained in Dulbecco's Modified Eagle's Medium (Invitrogen, USA) supplemented with 10 % fetal bovine serum (FBS, Gibco, USA), and A549 cells in Ham's F-12 Nutrient Mixture (Invitrogen, Carlsbad, CA, USA) supplemented with 10 % FBS. All the cell lines were passaged every 3-4 days and cultured in a 37 ^{o}C humidified incubator under an atmosphere of 5% CO_2 in air.

Cell imaging. Cells at a seeding density of 20,000 cells well^{-1} in μ-clear 96-well plate were incubated with medium containing **HXL2**@CD-AuNP (10 μM/100 nM) for 15 min at 37 ^{o}C with 5 % CO_2. Cell images were obtained by operetta (Perkin Elmer, USA) after washing with PBS. An excitation channel of 410-430 nm and emission channel of 460-540 nm were used. Data were mean of three independent experiments, and at least 500 cells for each condition were analyzed and plotted by Columbus analysis

system (Perkinelmer, US).

Cell viability. Cells at a seeding density of 8,000 cells well^{-1} in 96-well plate were incubated with medium containing different concentrations of HCPT and HCPT@nanocomposite for 15 min, and then cultured in growth medium for 72 h. The resulting cell viability was determined by MTS assay (CellTiter 96® AQ_{ueous} Assay).

Determination of reactive oxygen species (ROS). *Solution-based determination of ROS:* Dihydrorhodamine-123 (DHR123) was used as ROS tracking agent and protoporphyrin IX (PpIX) as a model photosensitizer. Oxidation of DHR123 by ROS resulted in the formation of fluorescent Rhodamine 123 (R123). **HXL2**@CD-AuNP (0.56 µM/7 nM) in the absence and presence of PNA (30 µM, aggregate), 6 µM DHR123, and 6 µM PpIX was maintained constant for all measurements. After irradiation at 600 nm, the fluorescence was measured on a Varian Cary Eclipse fluorescence spectrophotometer with an excitation of 485 nm. *Cell-based determination of (ROS):* Singlet Oxygen Sensor Green (SOSG) reagent was prepared at a final concentration of 4 µM DHR123 and 4 µM PpIX in PBS. Cells were incubated with the SOSG solution for 2 h after treating with HCPT and HCPT@nanocomposite. Then, cells in each well were refreshed with 100 µL PBS for light irradiation at 600 nm. After the irradiation, the well plate was further incubated for 20 min at 37 °C and then the fluorescence intensity was measured using a microplate reader with an excitation of 485 (emission: 528 nm).

Cellular photodynamic therapy. Cells were seeded at a density of 8,000 cells well^{-1} in 96-well plate prior to PDT. Cells were first incubated with HCPT@nanocomposite for 15 min and then irradiated with a broadband light source of a halogen lamp filtered through 600 nm filters for 30 min. After irradiation, cells were further cultured with complete medium for 72 h and the cell viability was determined by MTS assay (CellTiter 96® AQ_{ueous} Assay).

Real-time quantitative PCR. Total RNA was isolated from cells using TRIzol Reagent (Invitrogen) according to the manufacturer's protocol. Complementary DNA generated using a PrimeScript® RT reagent kit (TaKaRa, China) was analyzed by quantitative PCR using SYBR® Premix Ex TaqTM. Real-time quantitative PCR was performed using an Mx3005P PCR system (Agilent Technologies, USA). GAPDH was detected as the housekeeping gene. Primers used for qPCR analysis of human cell lines were as follows:

Human GAPDH forward, 5′-ATCACTGCCACCCAGAAGAC-3′

and reverse, 5′-ATGAGGTCCACCACCCTGTT-3′

Human ASGPR1 forward, 5′-CTGGACAATGAGGAGAGTGAC-3′
and reverse, 5′-TTGAAGCCCGTCTCGTAGTC-3′

Synthesis of b1 (Scheme S1) (a1[2] and c1[3] [shown in Scheme S3] were prepared according to previous protocols). Azide **b2** (452 mg, 0.98 mmol) was dissolved in CH_3OH (20 mL), followed by addition of $NH_3{\cdot}H_2O$ (1.3 mL, 5.8 mmol). The mixture was stirred over night at room temperature, and then concentrated in vacuum to give a crude product. The product was purified by column chromatography (CH_2Cl_2/MeOH = 17:1, v/v) to obtain **b1** as a white solid (261 mg, 91%). R_f = 0.31 (CH_2Cl_2/MeOH = 10:1, v/v).

^{1}H NMR (400 MHz, MeOD) δ 4.45 (d, J = 11.1 Hz, 1H), 3.87 (s, 2H), 3.79–3.67 (m, 3H), 3.63–3.57 (m, 2H), 3.49 (d, J = 5.5 Hz, 3H), 3.17–3.14 (m, 2H), 1.28 (t, J = 7.2 Hz, 2H); ^{13}C NMR (101 MHz, MeOD) δ 102.9, 75.9, 73.9, 73.6, 73.5, 72.4, 70.6, 69.8, 54.1, 18.9.

HRMS (ESI, m/z): $[M + H]^+$ calcd for $C_{10}H_{20}N_3O_7^+$ 294.1296, found 294.1235.

HPLC: t_R = 4.503 min of methanol (0.6 mL min^{-1}), purity 96.1%.

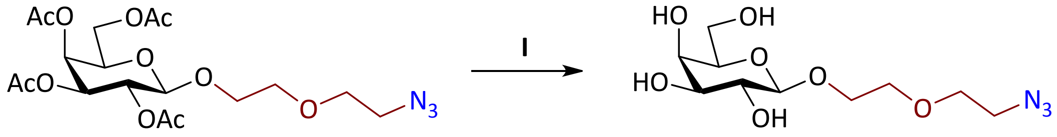

b2 **b1**

Scheme S1. Reagents and conditions: (I) $NH_3{\cdot}H_2O$, MeOH.

Synthesis of d1 (Scheme S2) (d3[4] was prepared according to a previous protocol). To a soln. of **d2** (198.7 mg, 1.02 mmol) in CH_2Cl_2 were added EDC (195.5 mg, 1.02 mmol) and HOBt (137.8 mg, 1.02 mmol). Then, **d3** (300 mg, 1.02 mmol) was added. The resulting mixture was stirred for 8 h under nitrogen. Then, the mixture was diluted with CH_2Cl_2 and washed with brine. The combined organic layer was dried over $MgSO_4$, filtered and concentrated in vacuum to give a crude product. The product was purified by column chromatography (CH_2Cl_2/MeOH = 40:1, v/v) to obtain **d1** (296.7 mg, 62%) as a yellow solid (296.7 mg, 62%). R_f = 0.69 (CH_2Cl_2/MeOH = 20:1, v/v).

^{1}H NMR (400 MHz, DMSO-d_6) δ 8.67–8.58 (m, 1H), 8.52–8.42 (m, 2H), 8.29 (d, J = 8.6 Hz, 1H), 7.95 (t, J = 4.9 Hz, 1H), 7.74–7.70 (m, 1H), 6.90 (d, J = 8.7 Hz, 1H), 4.74 (d, J = 2.2 Hz, 2H), 3.41–3.38 (m, 4H), 3.08 (t, J = 2.3 Hz, 1H), 1.95 (s, 2H), 1.76–1.73 (m, 4H), 1.64–1.61 (m, 5H), 1.39 (s, 6H); ^{13}C NMR (101 MHz, DMSO-d_6) δ 172.8, 136.0, 130.8, 130.3, 129.3, 128.1, 127.4(2), 127.2, 126.5, 126.1, 124.9, 124.7, 124.2, 124.1, 123.3, 72.9, 36.3(3), 33.1, 32.7, 31.9, 27.3(4), 26.7.

HRMS (ESI, m/z): $[M + H]^+$ calcd for $C_{29}H_{32}N_3O_3^+$ 470.2444, found 470.2421.

HPLC: t_R = 5.456 min of methanol (0.6 mL min^{-1}), purity 96.8%.

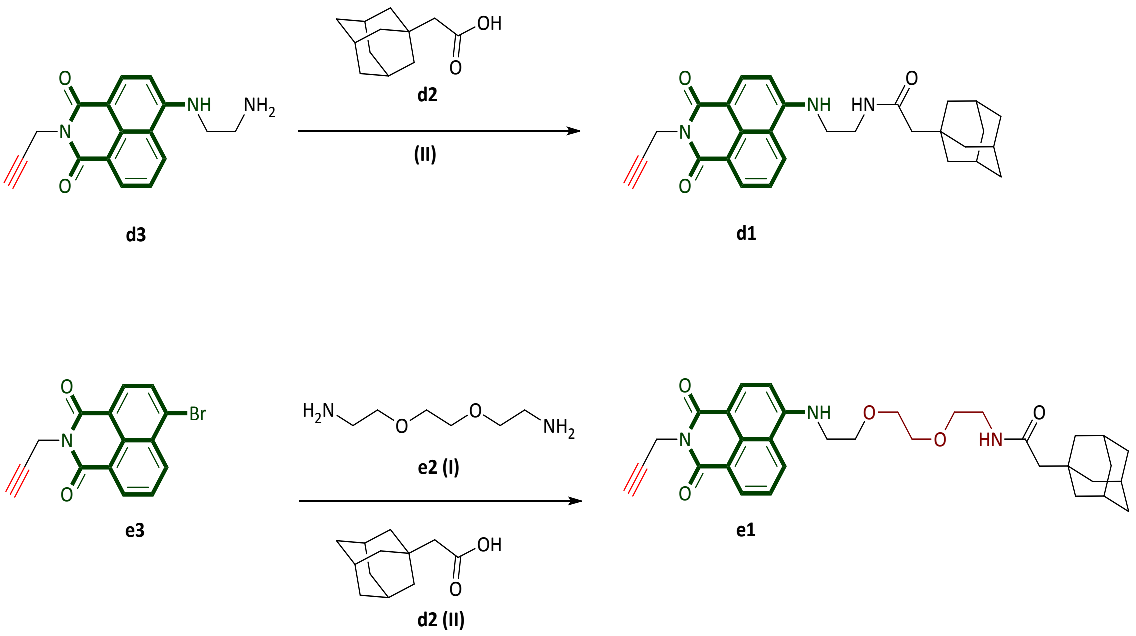

Scheme S2. Reagents and conditions: (I) Ethylene glycol monomethyl ether, CH_2Cl_2; (II) EDC/HOBt, CH_2Cl_2.

Synthesis of e1 (Scheme S2) (e2[5] and e3[6] were prepared according to previous protocols). To a soln. of **e3** (408.7 mg, 1.31 mmol) in CH_2Cl_2 were added **e2** (193.9 mg, 1.31 mmol) and ethylene glycol monomethyl ether (6 mL). The resulting mixture was stirred at room temperature for 5 h. Then, EDC (195.5 mg, 1.02 mmol) and HOBt (137.8 mg, 1.02 mmol) were directly added, followed by addition of **d2** (290.8 mg, 1.31 mmol). The resulting mixture was stirred for another 8 h under nitrogen. The mixture was then diluted with CH_2Cl_2 and washed with brine. The combined organic layer was dried over $MgSO_4$, filtered and concentrated in vacuum to give a crude product. The product was purified by column chromatography (CH_2Cl_2/MeOH = 30:1, v/v) to obtain **e1** (657 mg, 90%) as a yellow solid (657 mg, 90%). R_f = 0.1 (CH_2Cl_2/MeOH = 15:1, v/v).

^{1}H NMR (400 MHz, $CDCl_3$) δ 8.51–8.44 (m, 1H), 8.39–8.31 (m, 1H), 8.18 (s, 1H), 7.54 (d, J = 6.4 Hz, 1H), 6.60 (s, 1H), 6.10 (s, 1H), 4.92 (s, 2H), 3.89 (s, 2H), 3.71 (d, J = 11.0 Hz, 5H), 3.62–3.54 (m, 4H), 3.50–3.44 (m, 2H), 2.24 (t, J = 2.3 Hz, 1H), 1.90 (d, J = 4.0 Hz, 5H), 1.65 (d, J = 12.1 Hz, 2H), 1.55 (d, J = 10.3 Hz, 8H), 1.26–1.24 (m, 2H); ^{13}C NMR (101 MHz, DMSO-d_6) δ 169.9, 162.9, 161.9, 150.8, 134.3, 130.8, 129.3, 128.9, 127.7, 127.1, 124.3, 124.2, 121.4, 120.1, 119.1, 109.6, 107.1, 103.9, 79.8, 72.4, 69.7, 69.5, 69.1, 68.1, 49.9, 42.7, 41.9, 38.2, 36.4, 36.3, 32.1, 28.6, 27.9.

HRMS (ESI, *m/z*): $[M + H]^+$ calcd for $C_{33}H_{40}N_3O_5^+$ 558.2968, found 558.2915.

HPLC: t_R = 5.636 min of methanol (0.6 mL min^{-1}), purity 95.4%.

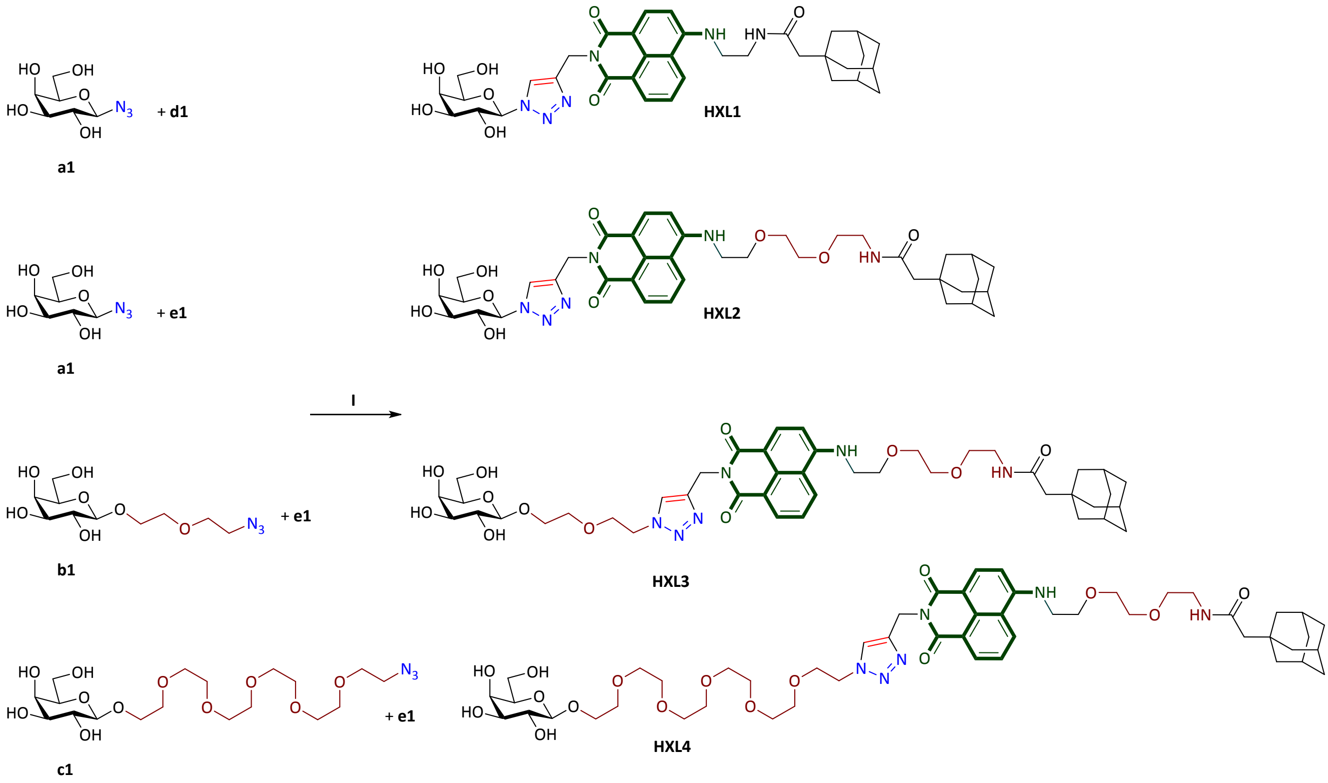

Scheme S3. Reagents and conditions: (I) $CuSO_4{\cdot}5H_2O$, Sodium ascorbate, $CH_2Cl_2/H_2O/t$-BuOH (2:1:1, v/v) at 60 °C.

General procedure for the click reaction (Scheme S3). To a soln. of azido glycoside and alkynyl lipid in a solvent mixture of $CH_2Cl_2/H_2O/t$-BuOH (2:1:1, v/v) were added $CuSO_4{\cdot}5H_2O$ and Na ascorbate. The mixture was stirred at 60 °C for 12 h under nitrogen. The resulting mixture was diluted with CH_2Cl_2 and washed with brine. The combined organic layer was dried over $MgSO_4$, filtered, and concentrated in vacuum to give a crude product, which was purified by column chromatography.

HXL1: From **a1** (100 mg, 0.48 mmol) and **d1** (228.8 mg, 0.48 mmol), column chromatography (CH_2Cl_2/MeOH = 8:1, v/v) afforded **HXL1** as a yellow solid (297.7 mg, 92%). R_f = 0.39 (CH_2Cl_2/MeOH = 5:1, v/v).

^{1}H NMR (400 MHz, DMSO-d_6) δ 8.67 (d, J = 8.4 Hz, 1H), 8.46 (d, J = 7.2 Hz, 1H), 8.29 (d, J = 8.5 Hz, 1H), 8.09 (s, 1H), 7.97 (t, J = 4.9 Hz, 1H), 7.78 (t, J = 5.3 Hz, 1H), 7.71 (t, J = 7.9 Hz, 1H), 6.89 (d, J = 8.7 Hz, 1H), 5.42 (d, J = 9.2 Hz, 1H), 5.29 (s, 2H), 5.20 (d, J = 6.0 Hz, 1H), 4.97 (d, J = 5.6 Hz, 1H), 4.71 (t, J = 5.8 Hz, 1H), 4.58 (d, J = 5.9 Hz, 1H), 3.96–3.93 (m, 1H), 3.70–3.67 (m, 1H), 3.65 (t, J = 6.0 Hz, 1H), 3.45–3.41 (m, 7H), 1.95 (s, 3H), 1.76 (d, J = 2.2 Hz, 6H), 1.65–1.62 (m, 6H), 1.24

(d, J = 11.1 Hz, 2H); ^{13}C NMR (101 MHz, DMSO-d_6) δ 177.8, 163.6, 162.6, 150.8, 134.3, 130.8, 129.5, 128.7, 124.3, 121.7, 120.1, 107.4, 106.3, 103.7, 88.0, 78.4, 73.6, 69.2, 68.3, 60.3, 42.7, 38.6(3), 37.2, 36.1(3), 34.8, 27.5(5).

HRMS (ESI, *m/z*): [M + H]$^+$ calcd for $C_{35}H_{43}N_6O_8^+$ 675.3142, found 675.3143.

HPLC: t_R = 4.28 min of methanol (0.6 mL min^{-1}), purity 94.9%.

HXL2: From **a1** (200 mg, 0.98 mmol) and **e1** (543.7 mg, 0.98 mmol), column chromatography (CH_2Cl_2/MeOH = 10:1, v/v) afforded **HXL2** as a yellow solid (709.4 mg, 95%). R_f = 0.65 (CH_2Cl_2/MeOH = 5:1, v/v).

^{1}H NMR (400 MHz, DMSO-d_6) δ 8.74 (d, J = 8.3 Hz, 1H), 8.44 (d, J = 7.2 Hz, 1H), 8.27 (d, J = 8.5 Hz, 1H), 8.08 (s, 1H), 7.89 (s, 1H), 7.69–7.65 (m, 2H), 6.83 (d, J = 8.7 Hz, 1H), 5.41 (d, J = 9.1 Hz, 1H), 5.27 (s, 2H), 5.20 (d, J = 5.9 Hz, 1H), 4.97 (d, J = 5.6 Hz, 1H), 4.72 (t, J = 5.6 Hz, 1H), 4.59 (d, J = 5.8 Hz, 1H), 3.99–3.90 (m, 1H), 3.71 (t, J = 5.2 Hz, 3H), 3.64 (t, J = 5.8 Hz, 1H), 3.57–3.54 (m, 4H), 3.53–3.45 (m, 6H), 3.15–3.12 (m, 3H), 1.85 (s, 3H), 1.78 (s, 2H), 1.58 (s, 2H), 1.51–1.49 (m, 8H), 1.21 (s, 2H); ^{13}C NMR (101 MHz, DMSO-d_6) δ 170.0, 163.5, 162.6, 150.8, 134.4, 130.9, 129.6, 124.3, 121.7, 120.2, 107.5, 103.9, 88.0, 78.4, 73.6, 69.7, 69.5, 69.2, 69.1, 68.4, 68.3, 68.1, 60.3, 49.9, 42.7, 42.0(3), 36.4(3), 34.8, 32.1(3), 27.9(3).

HRMS (ESI, *m/z*): [M + Na]$^+$ calcd for $C_{39}H_{50}N_6O_{10}^+$ 785.3486, found 785.3482.

HPLC: t_R = 5.07 min of methanol (0.6 mL min^{-1}), purity 95.2%.

HXL3: From **b1** (100 mg, 0.34 mmol) and **e1** (190.1 mg, 0.34 mmol), column chromatography (CH_2Cl_2/MeOH = 9:1, v/v) afforded **HXL3** as a yellow solid (263.1 mg, 91%). R_f = 0.68 (CH_2Cl_2/MeOH = 5:1, v/v).

^{1}H NMR (400 MHz, MeOD) δ 8.13 (t, J = 8.1 Hz, 2H), 8.01–7.89 (m, 2H), 7.34–7.24 (m, 1H), 6.43 (d, J = 8.7 Hz, 1H), 5.24 (s, 2H), 4.66 (s, 1H), 4.45 (t, J = 4.9 Hz, 2H), 3.81–3.78 (m, 6H), 3.68–3.59 (m, 5H), 3.58–3.52 (m, 3H), 3.46–3.38 (m, 8H), 3.24 (d, J = 5.5 Hz, 2H), 1.73 (s, 5H), 1.55–1.52 (m, 2H), 1.42 (d, J = 2.1 Hz, 7H), 1.19 (d, J = 9.9 Hz, 3H); ^{13}C NMR (101 MHz, MeOD) δ 173.9, 165.8, 165.1, 152.4, 135.9, 132.3, 130.9, 129.4, 126.1, 125.4, 122.9, 121.5, 109.3, 105.2, 101.7, 74.7, 72.5, 72.1, 71.6, 71.3, 71.1, 70.7, 70.3, 70.0, 68.6, 67.6, 62.8, 51.8, 51.4, 44.2, 43.6(3), 40.2, 37.8(3), 35.8, 33.7, 30.1(4).

HRMS (ESI, *m/z*): [M + Na]$^+$ calcd for $C_{43}H_{58}N_6O_{12}^+$ 873.4010, found 873.3985.

HPLC: t_R = 5.819 min of methanol (0.6 mL min^{-1}), purity 96.7%.

HXL4: From **c1** (140 mg, 0.29 mmol) and **e1** (166.3 mg, 0.29 mmol), column chromatography (CH_2Cl_2/MeOH = 8:1, v/v) afforded **HXL4** as a yellow solid (273.8 mg, 92%). R_f = 0.56 (CH_2Cl_2/MeOH = 4:1, v/v).

^{1}H NMR (400 MHz, MeOD) δ 8.33 (d, J = 8.0 Hz, 1H), 8.28 (d, J = 7.2 Hz, 1H), 8.11 (d, J = 8.6 Hz, 1H), 8.04 (s, 1H), 7.50–7.41 (m, 1H), 6.60 (d, J = 8.7 Hz, 1H), 5.34 (s, 2H), 4.59 (t, J = 4.8 Hz, 2H), 4.33 (t, J = 7.9 Hz, 1H), 4.00–3.97 (m, 1H), 3.89–3.86 (m, 2H), 3.84 (t, J = 5.5 Hz, 2H), 3.79–3.63 (m, 12H), 3.56–3.51 (m, 16H), 3.49 (d, J = 4.5 Hz, 2H), 3.35 (d, J = 3.6 Hz, 3H), 3.22–3.18 (m, 2H), 1.86 (s, 4H), 1.65 (d, J = 12.1 Hz, 3H), 1.58–1.50 (m, 7H), 1.34 (d, J = 7.3 Hz, 3H); ^{13}C NMR (101 MHz, MeOD) δ 173.8, 165.7, 164.9, 152.5, 135.8, 132.2, 130.9, 129.6, 126.2, 125.5, 122.9, 121.5, 109.2, 105.1, 104.1, 76.9, 74.8, 72.4, 71.6, 71.3, 71.2(2), 71.1, 70.9, 70.8, 70.7(2), 70.5, 70.4, 70.0, 69.1, 62.5, 51.8, 51.7, 51.3(2), 47.8, 44.3(2), 43.6, 40.1, 37.8(4), 35.8, 33.7, 30.1(4).

HRMS (ESI, *m/z*): $[M + H]^+$ calcd for $C_{51}H_{75}N_6O_{16}^+$ 1027.5240, found 1027.5200.

HPLC: t_R = 5.909 min of methanol (0.6 mL min^{-1}), purity 93.2%.

S3. Spectral copies of new compounds

^{1}H NMR of b1

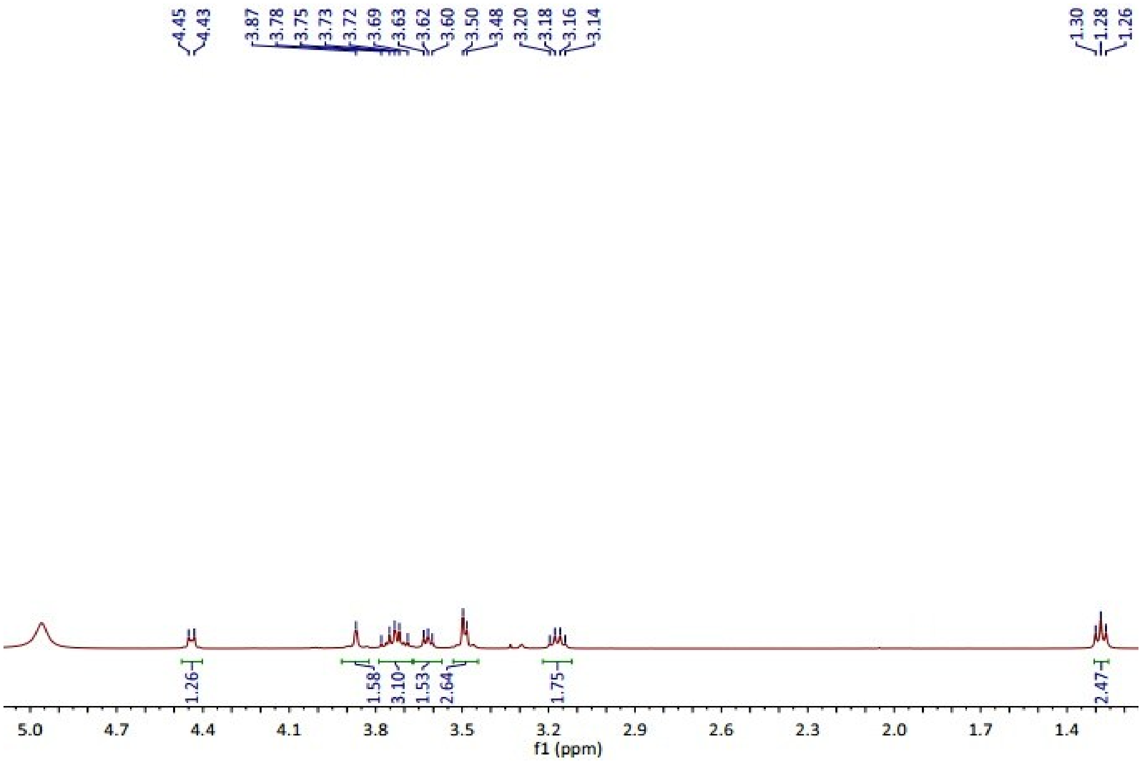

^{13}C NMR of b1

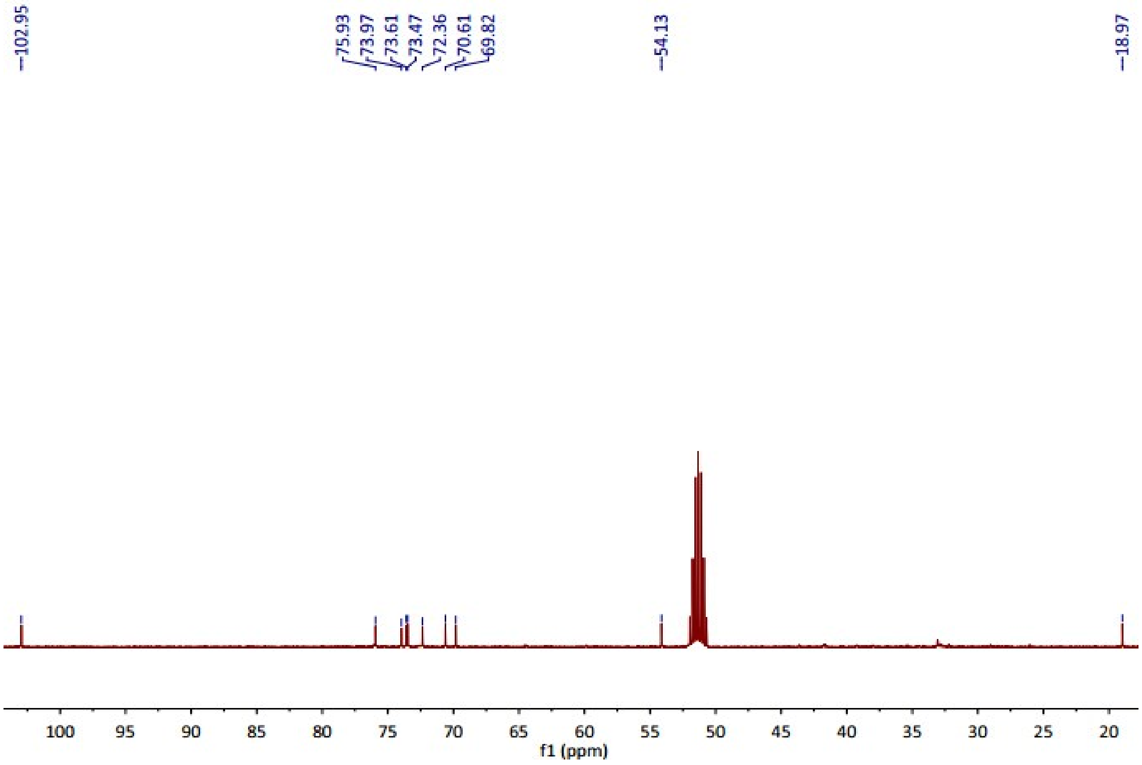

^{1}H NMR of d1

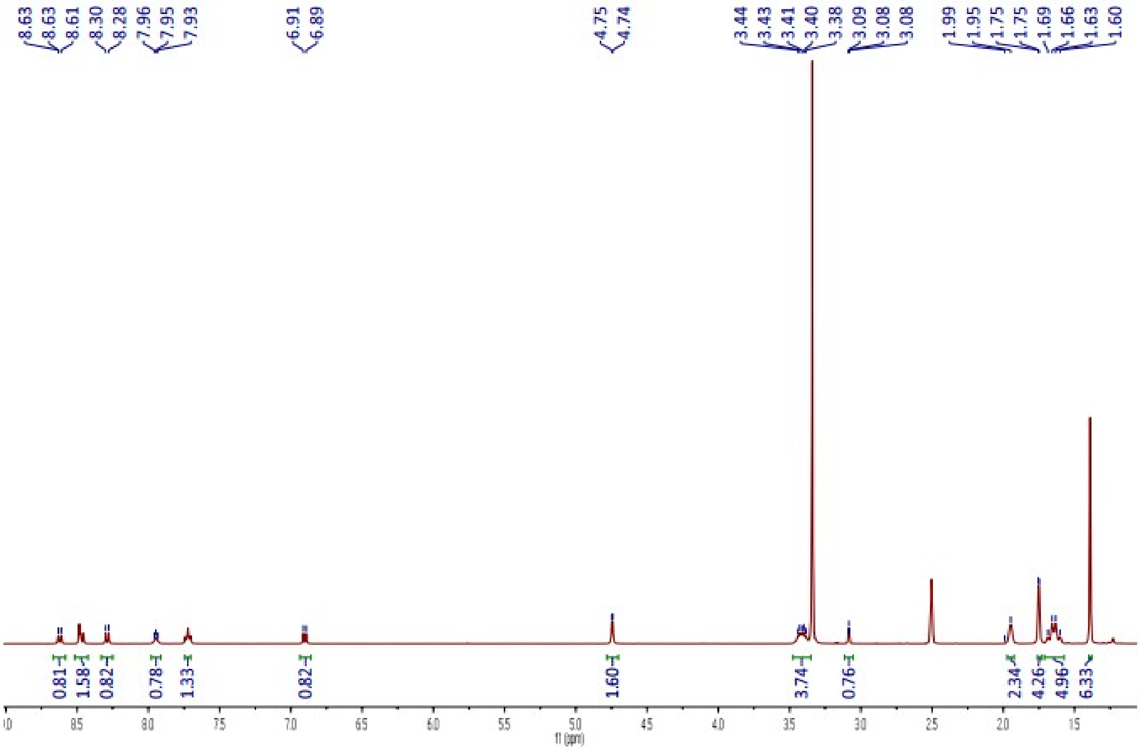

^{13}C NMR of d1

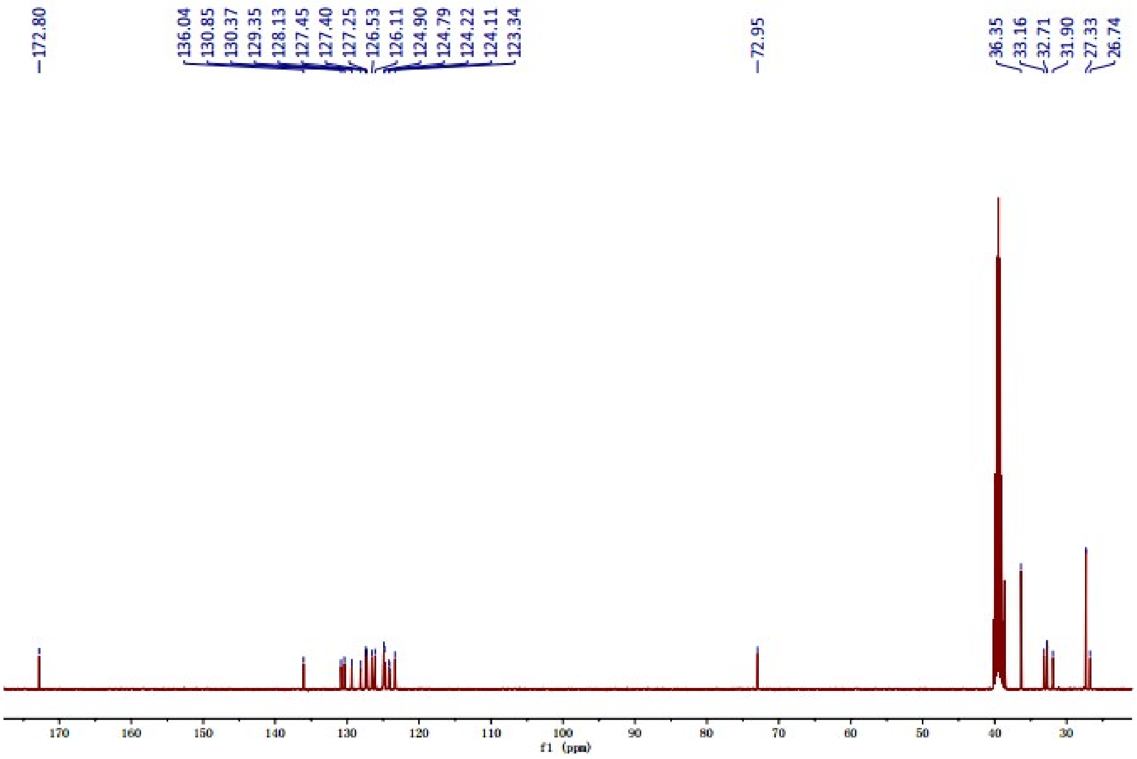

^{1}H NMR of e1

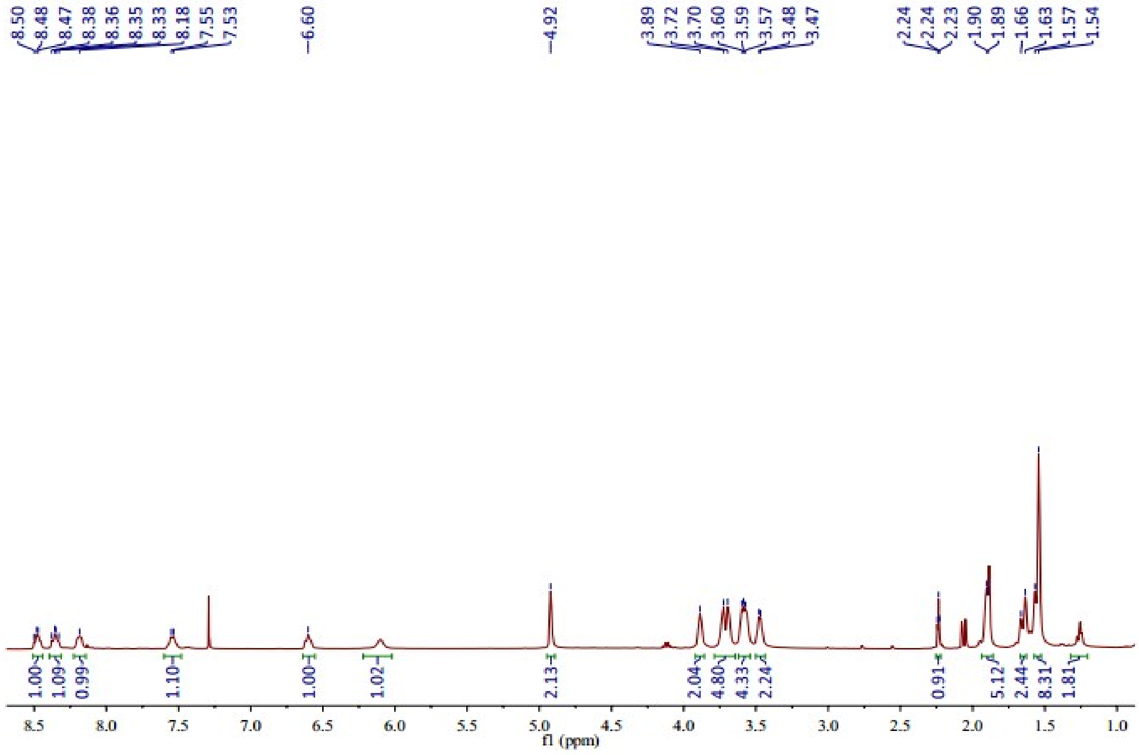

^{13}C NMR of e1

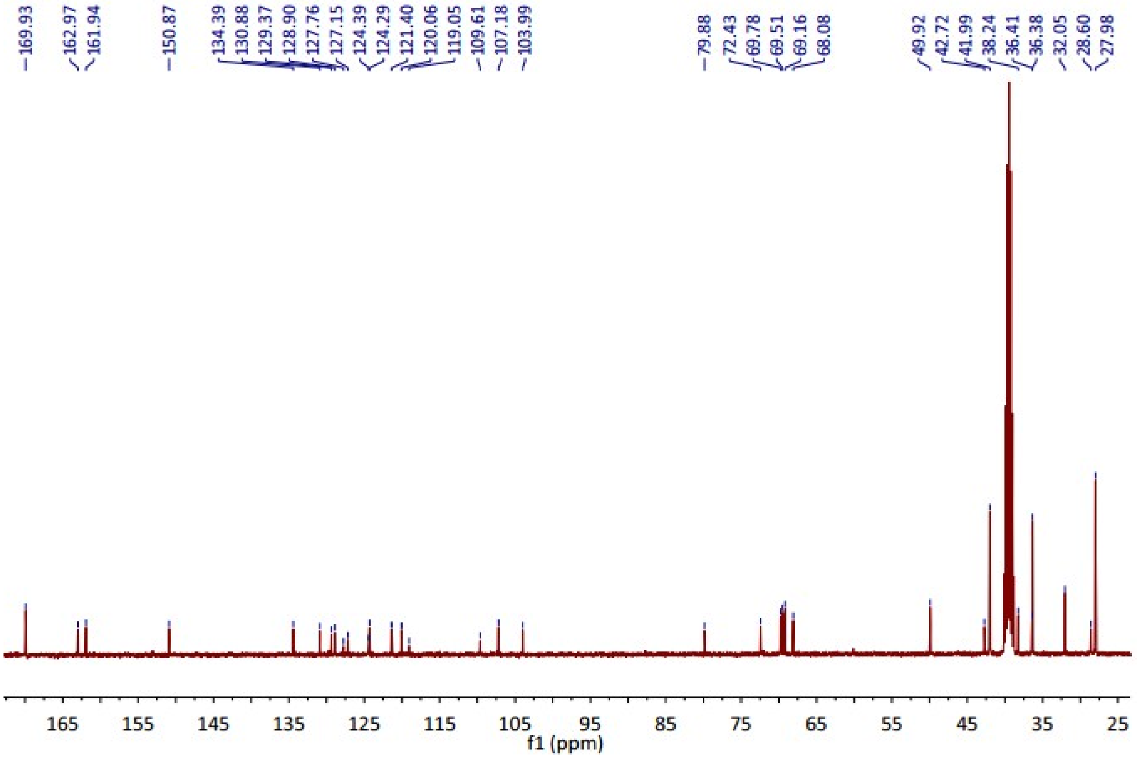

^{1}H NMR of HXL1

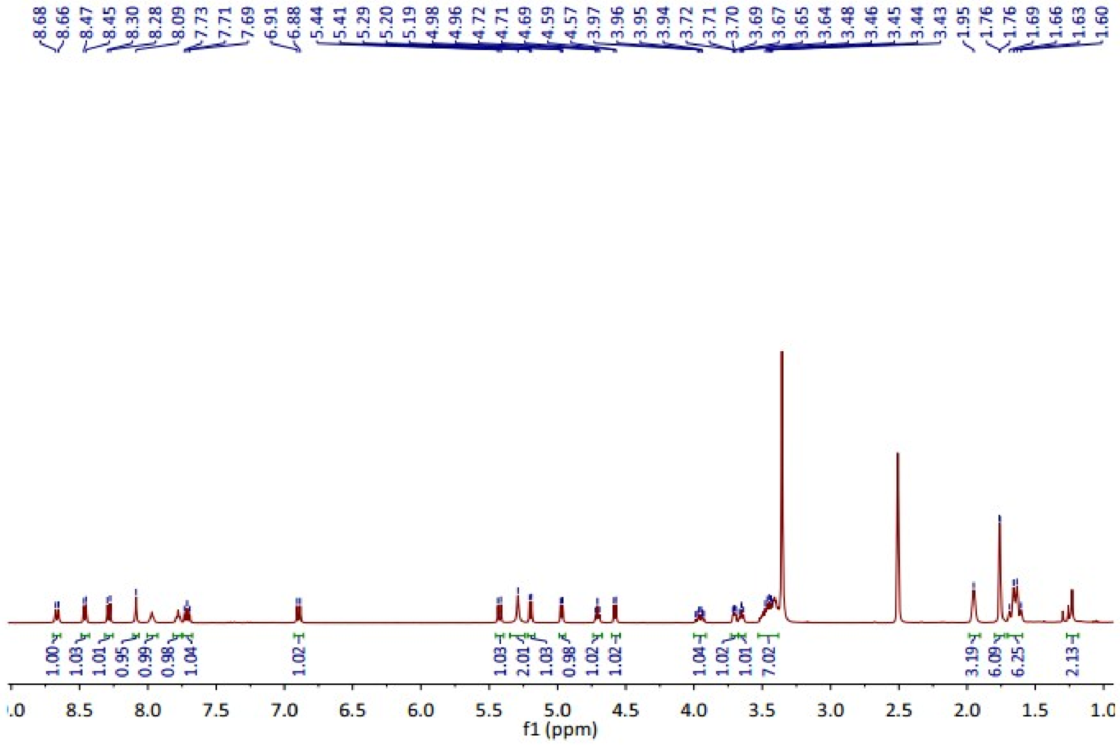

^{13}C NMR of HXL1

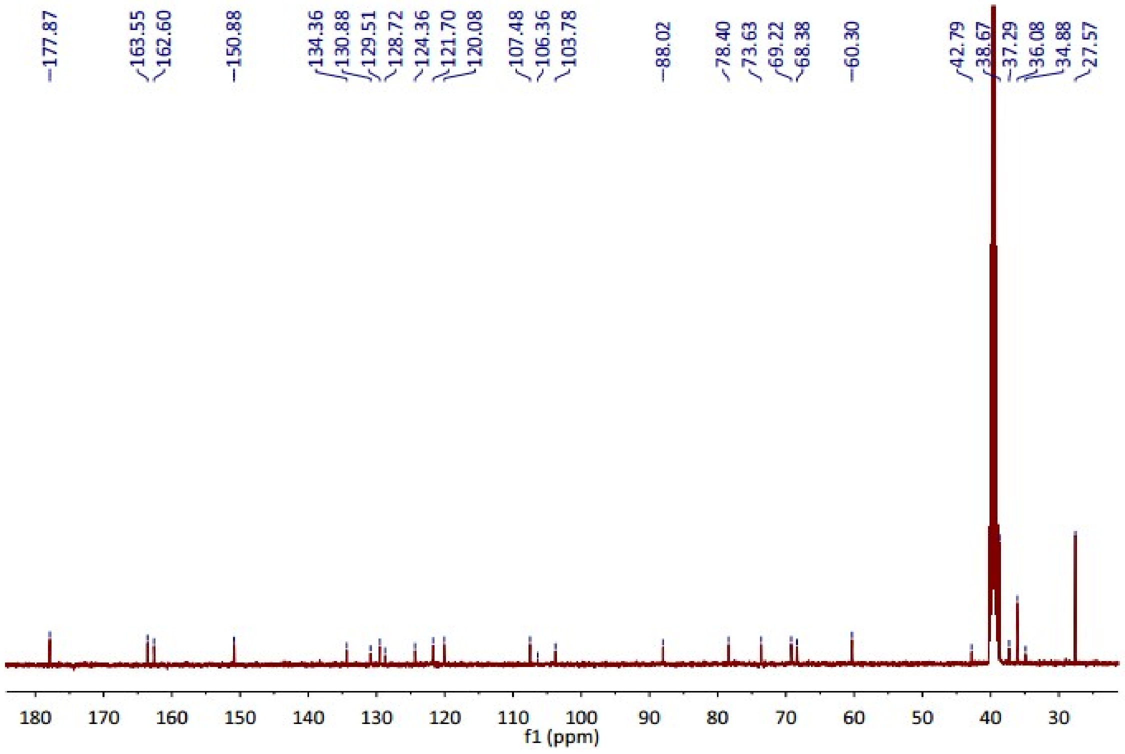

^{1}H NMR of HXL2

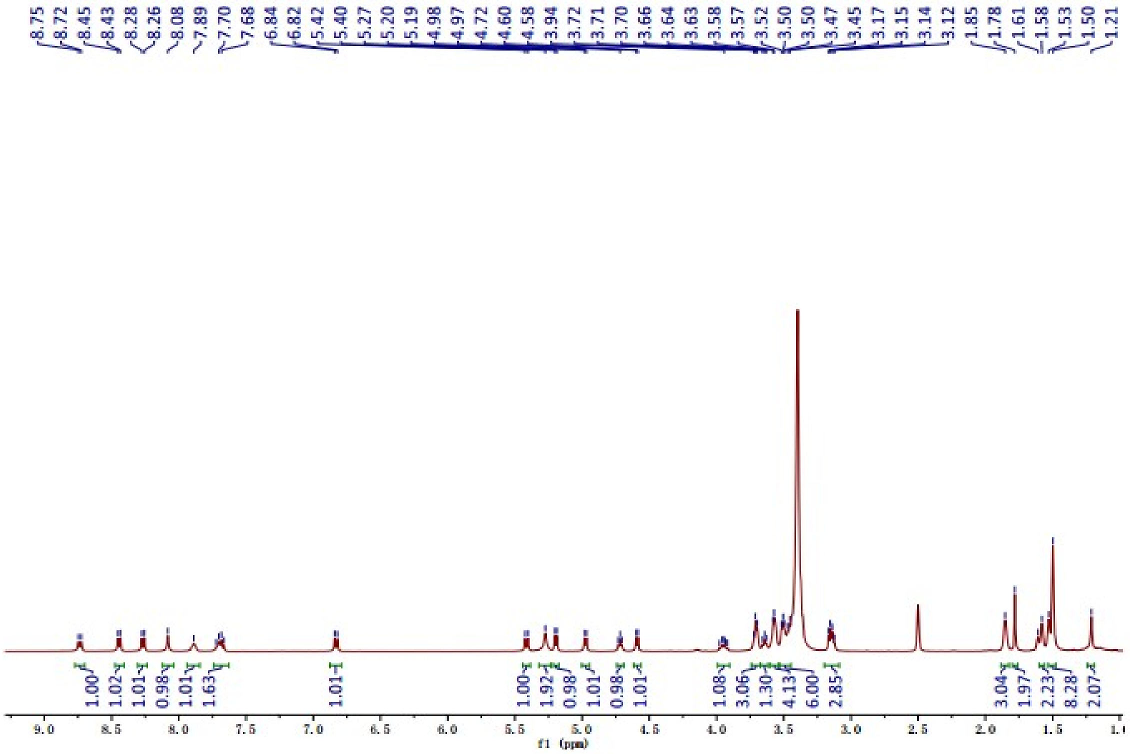

^{13}C NMR of HXL2

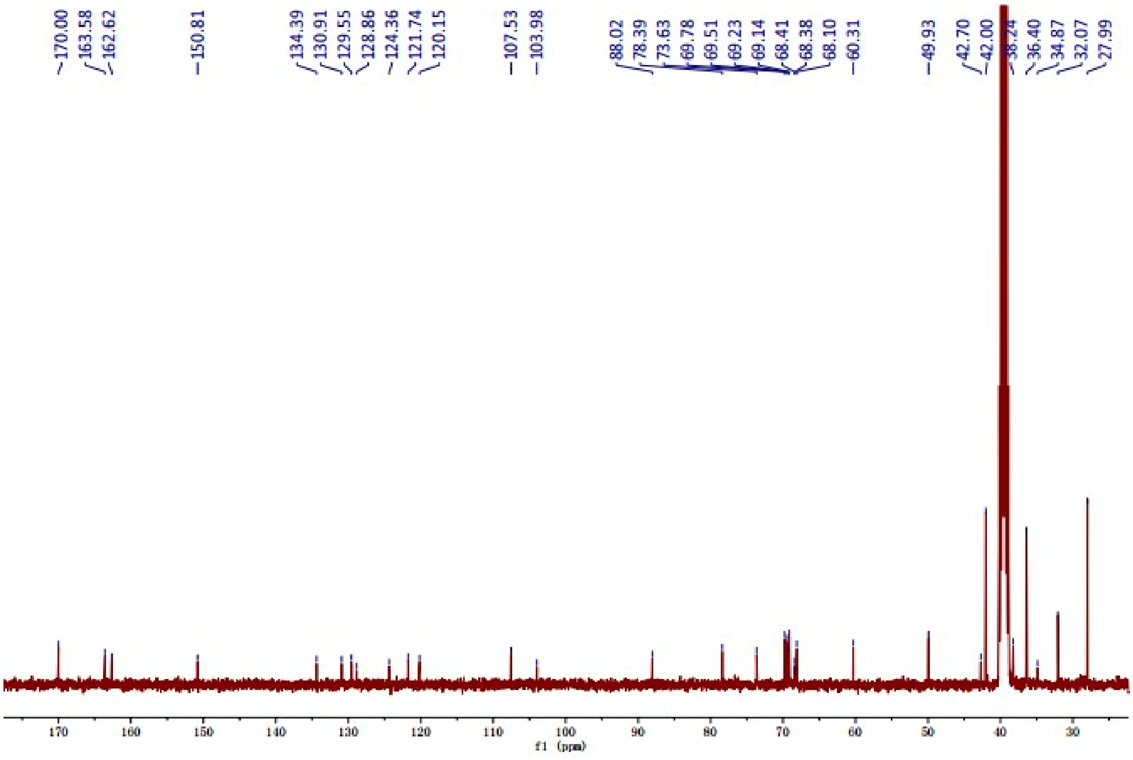

^{1}H NMR of HXL3

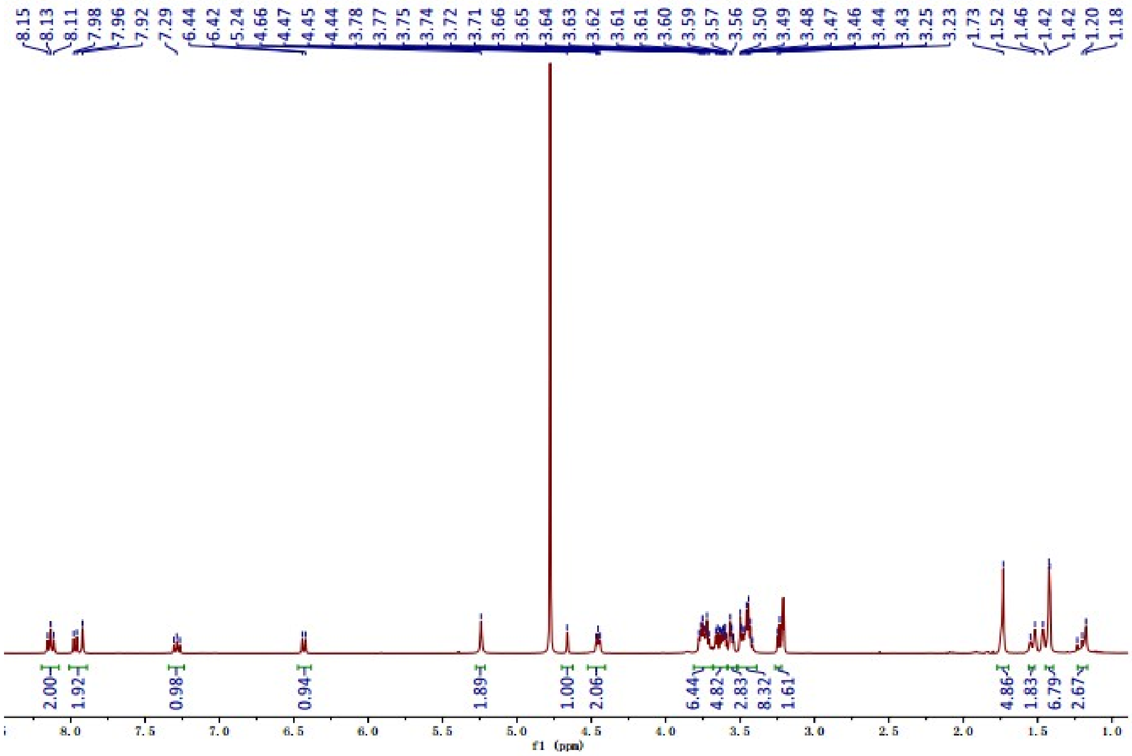

^{13}C NMR of HXL3

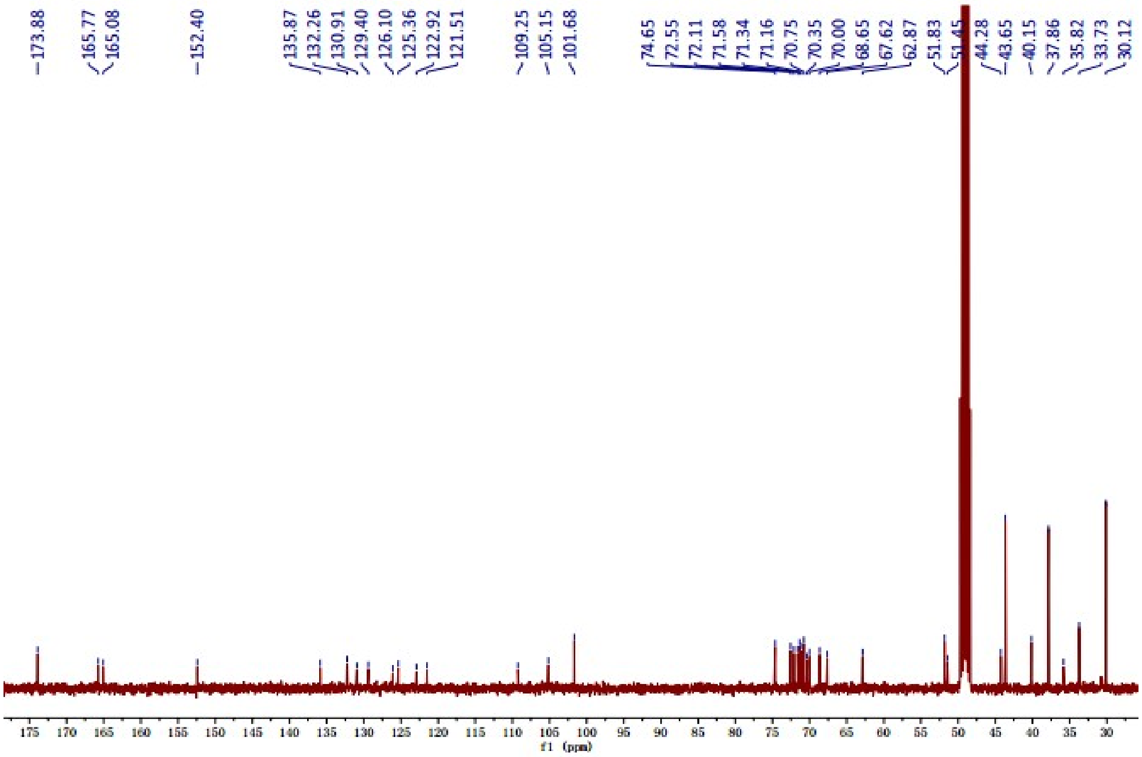

^{1}H NMR of HXL4

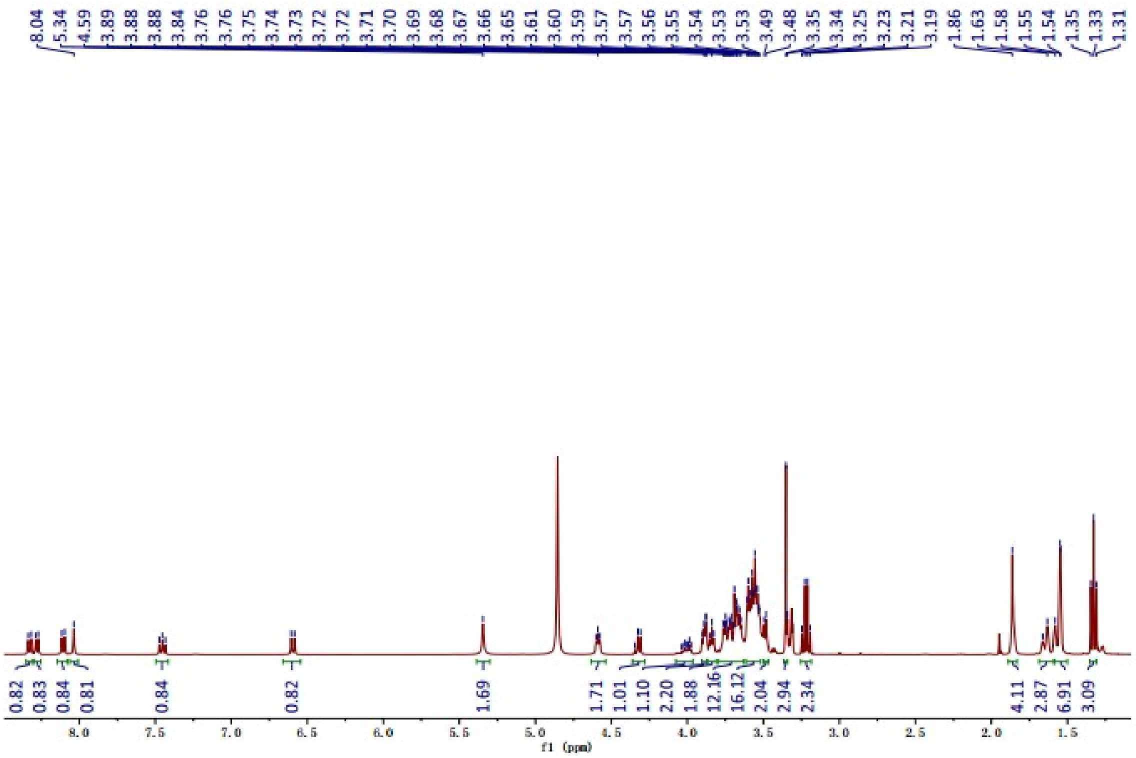

^{13}C NMR of HXL4

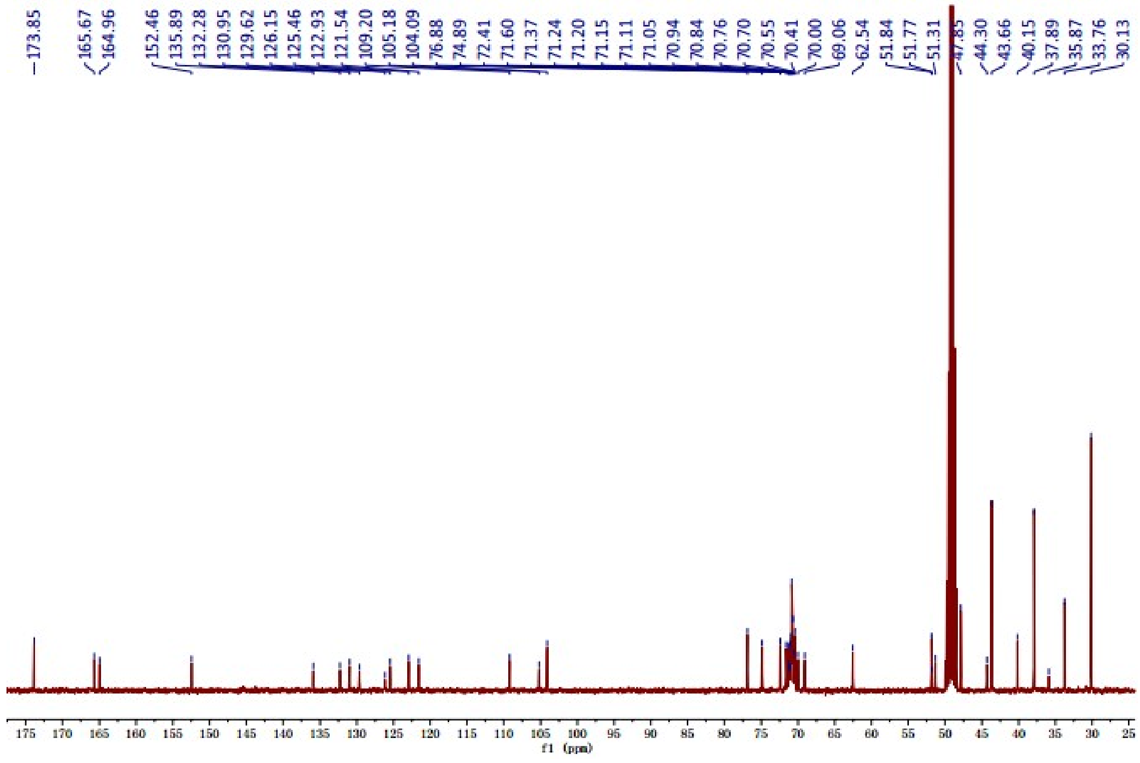

S4. Additional references

(1) Zhang, N.; Liu, Y.; Tong, L.; Xu, K.; Zhuo, L.; Tang, B. *Analyst* **2008**, *133*, 1176–1181.

(2) Hu, X.-L.; Li, D.; Shao, L.; Dong, X.; He, X.-P.; Chen, G.-R.; Chen, D. *ACS Med. Chem. Lett.* **2015**, *6*, 793−797.

(3) Mattarella, M.; Harties, G. J.; Wennekes, T.; Zuihof, H.; Sieqel, J. S. *Org. Biomol. Chem.* **2013**, *11*, 4333-4339.

(4) Yu, X.; Ge, X.; Lan, H.; Li, Y.; Geng, L.; Zhen, X.; Yi, T. *ACS Appl. Mater. Interface* **2015**, *7*, 24312−24321.

(5) Das, M.; Singh, R. P.; Datir, S. R.; Jain, S. *Bioconjugate Chem.* **2013**, *24*, 626−639.

(6) Ma, Z.; Zhang, P.; Yu, X.; Lan, H.; Li, Y.; Xie, D.; Li, J.; Yi, T. *J. Mater. Chem. B* **2015**, *3*, 7366–7371.
